# Prognostic accuracy of clinical markers of postpartum bleeding in predicting maternal mortality or severe morbidity: a WHO individual participant data meta-analysis

**DOI:** 10.1016/S0140-6736(25)01639-3

**Published:** 2025-10-25

**Authors:** Ioannis Gallos, Caitlin R Williams, Malcolm J Price, Aurelio Tobias, Adam Devall, John Allotey, Fernando Althabe, Jenny A Cresswell, Jill Durocher, A Metin Gülmezoglu, Christian Haslinger, Rodolfo C Pacagnella, Loïc Sentilhes, Soha Sobhy, Idnan Yunas, Jonathan J Deeks, Arri Coomarasamy, Olufemi T Oladapo, WHO Consortium on Postpartum Haemorrhage Definition, Anderson BOROVAC-PINHEIRO, Guillermo CARROLI, Arri COOMARASAMY, Jill DUROCHER, Fadhlun M. ALWY AL-BEITY, Sue FAWCUS, Mario FESTIN, Hadiza S. GALADANCI, Shivaprasad GOUDAR, A. Metin GÜLMEZOGLU, Christian HASLINGER, G. Justus HOFMEYR, Pisake LUMBIGANON, Kidza MUGERWA, Rodolfo C. PACAGNELLA, Zahida QURESHI, Loïc SENTILHES, Lumaan SHEIKH, John ALLOTEY, Arri COOMARASAMY, Adam DEVALL, Jonathan J. DEEKS, Malcolm PRICE, Soha SOBHY, Aurelio TOBIAS, Idnan YUNAS, Fernando ALTHABE, Jenny CRESSWELL, Ioannis GALLOS, Olufemi T. OLADAPO, Caitlin R. WILLIAMS

**Affiliations:** o University of Campinas, São Paulo, Brazil; p Centro Rosarino de Estudios Perinatales, Rosario, Argentina; q Tommy's National Centre for Miscarriage Research, University of Birmingham, UK; r Gynuity Health Projects, New York, USA; s Muhimbili University of Health and Allied Sciences, Dar es Salaam, Tanzania; t University of Cape Town, South Africa; u University of the Philippines, Manila, The Philippines; v Bayero University, Kano, Nigeria; w Women's and Children's Health Research Unit, Jawaharlal Nehru Medical College, KLE Academy of Higher Education and Research, Belagavi, India; x Concept Foundation, Geneva, Switzerland; y Department of Obstetrics, University Hospital Zurich, University of Zurich, Switzerland; z Effective Care Research Unit, University of the Witwatersrand, Walter Sisulu University, East London, South Africa; aa Faculty of Medicine, Khon Kaen University, Thailand; ab Makerere University, Kampala, Uganda; ac University of Campinas, São Paulo, Brazil; ad University of Nairobi, Nairobi, Kenya; ae Department of Obstetrics and Gynecology, Bordeaux University Hospital, France; af Aga Khan University, Karachi, Pakistan; ag Institute of Life Course and Medical Sciences, University of Liverpool, Liverpool, UK; ah Tommy's National Centre for Miscarriage Research, University of Birmingham, UK; ai Tommy's National Centre for Miscarriage Research, University of Birmingham, UK; aj Department of Applied Health Sciences, University of Birmingham, Birmingham, UK; ak Department of Public Health, Canadian University Dubai, Dubai, United Arab Emirates; al Tommy's National Centre for Miscarriage Research, University of Birmingham, UK; am Spanish Council for Scientific Research, Barcelona, Spain; an Department of Metabolism and Systems Science, College of Medicine and Health, University of Birmingham, Birmingham, UK; ao UNDP-UNFPA-UNICEF-WHO-World Bank Special Program of Research, Development, and Research Training in Human Reproduction, Department of Sexual and Reproductive Health and Research, WHO, Geneva, Switzerland; ap UNDP-UNFPA-UNICEF-WHO-World Bank Special Program of Research, Development, and Research Training in Human Reproduction, Department of Sexual and Reproductive Health and Research, WHO, Geneva, Switzerland; aq UNDP-UNFPA-UNICEF-WHO-World Bank Special Program of Research, Development, and Research Training in Human Reproduction, Department of Sexual and Reproductive Health and Research, WHO, Geneva, Switzerland; ar UNDP-UNFPA-UNICEF-WHO-World Bank Special Program of Research, Development, and Research Training in Human Reproduction, Department of Sexual and Reproductive Health and Research, WHO, Geneva, Switzerland; as UNDP-UNFPA-UNICEF-WHO-World Bank Special Program of Research, Development, and Research Training in Human Reproduction, Department of Sexual and Reproductive Health and Research, WHO, Geneva, Switzerland; a UNDP/UNFPA/UNICEF/WHO/World Bank Special Programme of Research, Development and Research Training in Human Reproduction, Department of Sexual, Reproductive, Maternal, Child, Adolescent Health and Ageing, WHO, Geneva, Switzerland; b Department of Public Health, Canadian University Dubai, Dubai, United Arab Emirates; c Spanish Council for Scientific Research, Barcelona, Spain; d Tommy's National Centre for Miscarriage Research, Institute of Metabolism and Systems Research, University of Birmingham, Birmingham, UK; e Institute of Life Course and Medical Sciences, University of Liverpool, Liverpool, UK; f Gynuity Health Projects, New York, NY, USA; g Concept Foundation, Geneva, Switzerland; h Department of Obstetrics, University Hospital Zurich, University of Zurich, Zurich, Switzerland; i Department of Obstetrics and Gynecology, School of Medical Sciences, University of Campinas, São Paulo, Brazil; j Department of Obstetrics and Gynecology, Bordeaux University Hospital, Bordeaux, France; k Nuffield Department of Women's and Reproductive Health, University of Oxford, Oxford, UK; l Department of Metabolism and Systems Science, College of Medicine and Health, University of Birmingham, Birmingham, UK; m Department of Applied Health Sciences, University of Birmingham, Birmingham, UK; n NIHR Birmingham Biomedical Research Centre, hosted by University Hospitals Birmingham NHS Foundation Trust in partnership with the University of Birmingham, Birmingham, UK

## Abstract

**Background:**

Postpartum haemorrhage (excessive bleeding after birth) is a leading cause of maternal mortality and morbidity worldwide. However, there is no global consensus on which clinical markers best define excessive bleeding or reliably predict adverse maternal outcomes. The aim of this study was to assess the prognostic accuracy of clinical markers of postpartum bleeding in predicting maternal mortality or severe morbidity.

**Methods:**

In this individual participant data meta-analysis, eligible datasets were identified through a global call for data issued by WHO and systematic searches of PubMed, MEDLINE, Embase, the Cochrane Library, and WHO trial registries (from database inception to Nov 6, 2024). Studies were eligible if they included at least 200 participants with objectively measured blood loss or other clinical markers of haemodynamic instability, and reported at least one clinical outcome of interest. Individual participant data were requested for all eligible studies. For each dataset, we computed the prognostic accuracy of each clinical marker to predict a composite outcome of maternal mortality or severe morbidity (blood transfusion, surgical interventions, or admission to intensive care unit). Five clinical markers were assessed: measured blood loss, pulse rate, systolic blood pressure, diastolic blood pressure, and shock index. Results were meta-analysed through two-level mixed-effects logistic regression models, with a bivariate normal model used to generate summary accuracy estimates. Clinical marker and threshold selections were informed by a WHO expert consensus process, which placed emphasis on maximising prognostic sensitivity (preferably >80%) over prognostic specificity (preferably ≥50%). This meta-analysis was registered on PROSPERO (CRD420251034918).

**Findings:**

We identified 33 potentially eligible datasets and successfully obtained and analysed full data for 12 datasets, comprising 312 151 women. At the conventional threshold of 500 mL, measured blood loss had a summary prognostic sensitivity of 75·7% (95% CI 60·3–86·4) and specificity of 81·4% (95% CI 70·7–88·8) for predicting the composite outcome. The preferred sensitivity threshold was reached at 300 mL (83·9% [95% CI 72·8–91·1]), although at the expense of reduced specificity (54·8% [95% CI 38·0–70·5]). Prognostic performance improved with a decision rule that combined the use of either blood loss thresholds less than 500 mL (≥300 mL to ≥450 mL) and any abnormal haemodynamic sign (pulse rate >100 beats per min, systolic blood pressure <100 mm Hg, diastolic blood pressure <60 mm Hg, or shock index >1·0) or 500 mL or more of blood loss, with sensitivities ranging from 86·9% to 87·9% and specificities from 66·6% to 76·1%.

**Interpretation:**

Measured blood loss below the conventional threshold, combined with abnormal haemodynamic signs, accurately predicts women at risk of death or life-threatening complications from postpartum bleeding and could support earlier postpartum haemorrhage diagnosis and treatment.

**Funding:**

The Gates Foundation and UNDP/UNFPA/UNICEF/WHO/World Bank Special Programme of Research, Development and Research Training in Human Reproduction.

## Introduction

Globally, an estimated 260 000 women die each year from complications of pregnancy and childbirth.^1^ Postpartum haemorrhage or excessive bleeding after birth, which affects millions of women annually, remains one of the leading causes of these deaths.^2^, ^3^ Although deaths related to postpartum haemorrhage have been nearly eliminated in high-income countries, they remain disproportionately high in low-income and lower-middle-income countries, particularly in sub-Saharan Africa and south Asia.^3^ The inequities in access to effective prevention and treatment strategies to avoid postpartum haemorrhage deaths are a stark reminder of the unfinished global agenda on maternal health and rights.

**Research in context**

**Evidence before this study**

Postpartum haemorrhage remains a leading cause of maternal mortality globally, despite the availability of effective treatments. Early identification of women at risk of life-threatening complications is essential to enable timely intervention with a first-response treatment bundle to avert death or severe morbidity. In clinical care, common markers of postpartum bleeding include volume of blood loss and changes in vital signs, such as pulse rate, blood pressure, and indices that combine these signs of haemodynamic changes (eg, shock index). However, there is no consensus on what clinical markers constitute excessive bleeding or which thresholds best predict serious maternal complications from postpartum bleeding. Available evidence on prognostic accuracy of these clinical markers is generally scarce, fragmented, and primarily derived from small, single-centre studies with heterogeneous methodologies. For example, blood loss is often underestimated in most clinical settings, and its sensitivity for detecting severe maternal outcomes varies widely depending on the threshold used and context. Similarly, although abnormal vital signs (eg, tachycardia, hypotension, and elevated shock index) are used to infer clinical deterioration, there is no clear evidence on which of these markers or their thresholds best predict maternal death or severe morbidity from postpartum bleeding. Systematic searches of PubMed, MEDLINE, Embase, the Cochrane Library, and WHO trial registries from inception to Nov 6, 2024, did not find any study that synthesised individual-level data across diverse populations to assess and compare the prognostic performance of these markers or established optimal thresholds for identifying women at highest risk of death or life-threatening complications.

**Added value of this study**

This study presents the most comprehensive prognostic analysis of postpartum haemorrhage data to date, incorporating individual-level data from 312 151 women across 12 datasets from 23 countries and diverse care settings. By using individual participant data, the study offers robust estimates of prognostic accuracy of commonly used clinical markers (measured blood loss, pulse rate, systolic and diastolic blood pressure, and shock index) for predicting maternal death or severe morbidity. It is the first analysis to systematically assess the prognostic accuracy of these markers and their thresholds, both individually and in combination. The findings show that measured blood loss thresholds lower than the conventional 500 mL (eg, ≥300 mL) when combined with any abnormal haemodynamic signs provide greater sensitivity and acceptable specificity for identifying women at risk of death or severe morbidity. Prognostic accuracy improved further when analyses were restricted to women with vaginal births, whereas accuracy was lower for the caesarean birth population based on data from a single study, limiting confidence in findings for this subgroup. No substantial subgroup differences were observed by country income level and individual baseline risk of postpartum haemorrhage.

**Implications of all the available evidence**

Findings from this study support the use of measured blood loss thresholds of less than 500 mL (eg, 300 mL or more) in combination with abnormal haemodynamic signs or measured blood loss threshold of 500 mL or more (whichever occurs first) as a more accurate approach for early identification of women at risk of life-threatening postpartum haemorrhage. These diagnostic criteria have the potential to improve early diagnosis and timely intervention, as part of efforts to reduce maternal mortality and morbidity associated with postpartum haemorrhage. These results provide an evidence base and a strong rationale for revisiting the clinical practice and policy definitions of postpartum haemorrhage.

Although some bleeding after birth is expected, there is no consensus on how much bleeding constitutes postpartum haemorrhage.^4^, ^5^ Contemporary clinical practice tends to rely on standardised volumetric thresholds of blood loss (typically ≥500 mL for postpartum haemorrhage and ≥1000 mL for severe postpartum haemorrhage) to define abnormal postpartum bleeding.^6^ However, these thresholds might not accurately reflect clinical risk, as individual tolerance to blood loss varies depending on factors such as circulating blood volume, haemoglobin concentration, and overall health status. These physiological factors are shaped by social determinants of health, including nutrition and access to care, and distribution of these determinants often exhibits social gradients.^7^ Consequently, a given volume of blood loss could be well tolerated by some women but catastrophic for others.

To reduce preventable deaths and severe morbidity from postpartum haemorrhage, there is a need for more accurate diagnostic criteria to identify women at the highest risk of severe complications. Identifying effective prognostic clinical markers would enable earlier diagnosis and timely intervention, particularly in low-resource settings where delays in postpartum haemorrhage care are common. The aim of this study was to assess the prognostic accuracy of clinical markers of postpartum bleeding in predicting maternal mortality or severe morbidity in women giving birth.

## Methods

### Search strategy and selection criteria

We conducted an individual participant data (IPD) meta-analysis. Given the type of data required for assessing prognostic accuracy, a synthesis of aggregate data was considered unlikely to be informative and an IPD meta-analysis was pursued instead.^8^ The IPD approach enabled detailed exploration of multiple thresholds and combinations of clinical markers across diverse populations, subgroups, and care settings.

Eligible studies were identified through a global call for data issued by WHO and systematic searches of PubMed, MEDLINE, Embase, the Cochrane Library, and WHO trial registries from database inception to Nov 6, 2024. Search terms included “pregnancy”, “birth”, and “postpartum haemorrhage” for the populations of interest combined with clinical markers (ie, blood loss, pulse, blood pressure, shock index, haemoglobin, and lactate; full strategy is in the appendix p 4). No language restrictions were applied. We screened reference lists of eligible studies and searched conference proceedings for additional unpublished data.

Studies were eligible if they included cohorts of participants with prognostic accuracy data (including both predictor and response variables) regardless of the study design, either observational or experimental. Predictor variables had to include data on objectively measured blood loss (by weighing blood or through tools for volumetric assessment, such as calibrated drapes or measuring jars) or on objectively assessed clinical markers, such as pulse rate, respiratory rate, blood pressure, uterine tone, or any clinical signs of haemodynamic changes. Studies that relied solely on visually estimated blood loss were excluded due to the known inaccuracy of this method. Response variable data had to include at least one of the following clinical outcomes: all-cause maternal mortality, transfusion of whole blood or blood products, need for mechanical or surgical intervention to stop the bleeding (eg, uterine tamponade, laparotomy, vessel ligation, compression sutures, interventional radiology, or hysterectomy), maternal sepsis, organ failure, admission to high-dependency or intensive care unit, or severe postnatal anaemia (<80 g/L). Datasets also had to contain data from at least 200 women, not be composed exclusively of women already diagnosed with postpartum haemorrhage, and contain data collected with a standardised protocol or through clinical or public health-care encounters after 1990. Eligible studies must have had previous ethics committee approval and the data holder had to be able to enter into a legal data sharing agreement with WHO.

Two authors (JA and SS) screened search results for potentially eligible studies and disagreements were resolved by discussion and consensus between the two authors. Authors of potentially eligible studies were invited by email (with up to three reminders) to respond to the WHO call for data. IPD were requested for all eligible studies, and principal investigators and their institutions were invited to contribute data via a secure WHO data portal and sign standard data sharing agreements. Data sharing agreements covered permissible data uses, access, and publication rights. Each data provider was asked to supply raw datasets, accompanying data dictionaries, original study protocols, and documentation of measurement methods. The acquired de-identified data were stored on an encrypted, access-restricted server at WHO headquarters, with audit trails for data access and modifications (appendix pp 5–6, 18–21). Two authors (IG and CRW) independently assessed eligibility of studies, and any disagreements were resolved by discussion and consensus between the two authors.

Institutional review board approval was not required for this study because only previously collected, de-identified data were used. All contributing studies had received appropriate ethical approvals, and no new participant recruitment or data collection was done for the current study. The methods followed a prespecified protocol, which was registered with PROSPERO (CRD420251034918) and made publicly available via the WHO website. Minor deviations from the protocol and their justifications are described in the appendix (p 11). We report this IPD meta-analysis in accordance with the PRISMA-IPD guidelines; a completed checklist is provided in the appendix (pp 44–47).

### Outcomes

The statistical outcomes of interest were measures of prognostic accuracy, including sensitivity, specificity, diagnostic odds ratio (DOR), and positive and negative likelihood ratios by selected thresholds for each clinical marker (appendix pp 8–9). The prognostic outcome of interest was a composite outcome defined as maternal death or any of the following severe morbidities: transfusion of whole blood or blood products; mechanical or surgical interventions to control bleeding (uterine tamponade, laparotomy, compression sutures, hysterectomy, uterine artery ligation, or interventional radiology); or admission to an intensive care or high-dependency unit.

### Data processing and analysis

We implemented a rigorous, prespecified protocol for data collection, processing, recoding, cleaning, quality checking, querying, and merging to ensure the integrity and high quality of the final dataset (appendix pp 5–8).

Briefly, the variables in each eligible dataset were mapped against prespecified variables in the IPD protocol and crosschecked for accuracy, validity, and internal consistency against the original study protocols and published or unpublished reports. Individual participant data were extracted on maternal characteristics (age, parity, gestational age, mode of birth, induction or augmentation of labour, duration of labour, episiotomy, perineal tear, retained placenta, birthweight, and use of prophylactic uterotonics), clinical markers of postpartum bleeding (measured blood loss, pulse rate, systolic and diastolic blood pressure, and shock index), and outcome data. The outcome was a composite of maternal death or life-threatening morbidity, including transfusion, major surgery, and admission to intensive or high-dependency care. Although data collection on maternal ethnicity was planned, none of the included studies reported this variable. De-identified individual participant data were harmonised and recoded through standard protocols. All datasets underwent systematic integrity assessment checks adapted from the Cochrane Pregnancy and Childbirth Trustworthiness Screening Tool.^9^

For clinical markers measured at several timepoints, we extracted values at defined intervals (15, 30, 45, and 60 min postpartum) and used the most abnormal value before diagnosis of postpartum haemorrhage for the primary analysis. For measured blood loss, we extracted the total blood loss at the time of diagnosis, if available, and the cumulative total at the end of the measurement. We extracted the target prognostic outcome of interest occurring after birth and until hospital discharge, irrespective of the cause. Available data were checked for consistency and completeness by at least two authors (CRW and IG). At least two of three authors (CRW, IG, or JA) independently assessed the risk of bias and concerns about applicability in the individual studies with the Quality Assessment of Prognostic Accuracy tool.^10^ Risk of bias assessments were independently done by authors who had no involvement in the primary studies. Conflicts were resolved through discussion and consensus among authors. We calculated the number of eligible studies and participants for which we obtained IPD from the total number of eligible studies and participants identified, to assess the degree of potential selection bias in the final analysis.

We conducted a complete case analysis of data. We used a two-stage approach to estimate prognostic accuracy measures, including sensitivities, specificities, DORs, and likelihood ratios with their 95% CIs for different thresholds of each clinical marker. In stage one, we calculated the prognostic accuracy estimates per study. In stage two, we applied a random-effects meta-analysis with a two-level mixed logistic regression model with independent binomial distributions for the true positives and true negatives in each study, and a bivariate normal model for computing the summary sensitivities and specificities across studies.^11^ We present the results in tables and graphs to show the individual study estimates, summary curve from the hierarchical summary receiver operating characteristic (ROC), a summary operating point (eg, summary values for sensitivity and specificity), 95% confidence region for the summary operating point, and 95% prediction region (ie, the confidence region for a forecast of the true sensitivity and specificity in a future study).

We calculated the prognostic accuracy measures at 50 mL incremental thresholds (above the median) for blood loss, at 10 beats per min for pulse rate, 10 mm Hg for systolic and diastolic blood pressure, and 0·1 unit for shock index. We considered a clinical marker as predictive when the DOR was greater than 2 for at least one threshold. We also examined the prognostic accuracy of three predefined decision rules combining clinical markers, to investigate whether combinations of markers improved accuracy compared with any individual marker alone. These rules were developed a priori based on clinical rationale, expert consensus, and literature review, following recommended principles for constructing composite rules to evaluate combinations of imperfect tests in diagnostic research.^12^ In the first decision rule, women were classified as at high risk of death or severe morbidity if any one marker was abnormal (eg, blood loss above the target threshold, pulse rate >100 beats per min (bpm) for tachycardia, systolic blood pressure <100 mm Hg or diastolic blood pressure <60 mm Hg for hypotension, or shock index score >1). In the second decision rule, women were classified as at high risk of death or severe morbidity if blood loss and at least one other clinical marker were abnormal. The third decision rule consisted of a composite point scoring system that assigned 1 point for blood loss thresholds lower than 500 mL, a maximum of 1 point for any other abnormal haemodynamic marker (pulse rate >100 bpm, systolic blood pressure <100 mm Hg, diastolic blood pressure <60 mm Hg, or shock index >1), and 2 points for blood loss of 500 mL or more. Women were classified as at high risk if their total score was 2 points or more.

We explored heterogeneity through subgroup analyses done with the bivariate model by mode of birth (vaginal *vs* caesarean), country income level (low and lower-middle income *vs* upper-middle and high income), and individual baseline postpartum haemorrhage risk (low *vs* high), when sufficient data for the subgroups were available. Participants were defined as having high baseline risk if they had any of the known risk factors, such as anaemia, grandmultiparity (five or more births), previous postpartum haemorrhage, previous caesarean birth, pre-eclampsia, a BMI of 30 kg/m^2^ or more, induction of labour, episiotomy, retained placenta, a child with a birthweight of 4500 g or more, multiple pregnancy, and assisted vaginal birth or caesarean birth. We explored the robustness of the findings through sensitivity analyses done by removing populations with potentially different prognoses from the analyses (ie, participants who had volumetric assessment of blood loss and participants who had different thresholds for initiating treatment [eg, ≥300 mL instead of the conventional threshold of ≥500 mL]). Subgroup and sensitivity results were examined by comparing the differences in DORs and prediction graphs in summary ROC plots.

A WHO technical consultation, involving 26 experts from all WHO regions, used a consensus-driven process to assess the relative importance of the prognostic sensitivity versus specificity of clinical markers in the context of predicting maternal death or severe morbidity from postpartum bleeding. There was consensus that experts would value sensitivity over specificity. Most experts (22 [85%] of 26) at the consultation indicated a preference for higher sensitivity, emphasising the importance of correctly identifying women at high risk of adverse outcomes to ensure timely intervention. A small proportion (one [4%] of 26) prioritised specificity, favouring the avoidance of unnecessary treatment in women not at risk, and three (12%) of 26 experts valued both equally. The experts prioritised prognostic sensitivity over specificity because first-response treatment of postpartum haemorrhage can prevent adverse outcomes; carries few risks; and is affordable, widely available, and easily implementable—unlike refractory postpartum haemorrhage treatment, which is costly, less accessible, and requires specialist care. Based on this consensus, clinical markers and thresholds were considered diagnostically valuable if they had a sensitivity exceeding 80% and a specificity of at least 50%.

We did all meta-analyses with metandi in Stata (version 17).

### Role of the funding source

The funders of this study had no role in study design, data collection, data analysis, data interpretation, or writing of this report.

## Results

**Figure 1:**
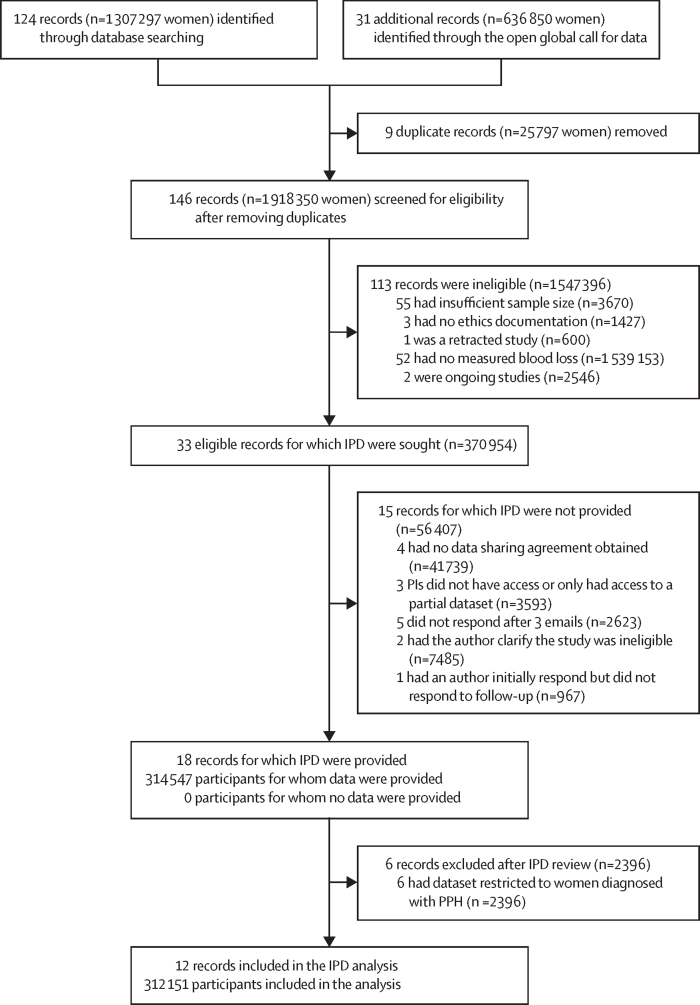
Flowchart of study selection IPD=individual participant data. PI=principal investigator. PPH=postpartum haemorrhage.

We identified 33 potentially eligible datasets, including a total of 370 954 women. IPD data were successfully obtained from 18 datasets (representing data from 314 547 women). 12 of these datasets were included in the final analysis, representing 312 151 (84·1%) of 370 954 potentially eligible participants (figure 1).^13^, ^14^, ^15^, ^16^, ^17^, ^18^, ^19^, ^20^, ^21^, ^22^, ^23^, ^24^ Four multicountry studies contributed more than 80% of all participants analysed. Three were individually randomised trials evaluating interventions to prevent postpartum haemorrhage,^16^, ^17^, ^24^ and the fourth was a cluster randomised trial that assessed the effectiveness of early diagnosis and treatment of postpartum haemorrhage.^15^ Most (306 109 [98·1%] of 312 047) women in the analysis gave birth vaginally. The rate of uterotonic prophylaxis was very high among study participants from studies that provided data on postpartum haemorrhage prophylaxis (105 046 [99·1%] of 106 041 women). Characteristics of the included studies and participants are provided in the appendix (pp 22–26). Overall, risk of bias and applicability concerns were low across studies (appendix p 27).

The final analysis evaluated five clinical markers (measured blood loss, pulse rate, systolic blood pressure, diastolic blood pressure, and shock index) for their accuracy to predict the composite outcome. All 12 datasets provided IPD on measured blood loss, with few missing data. Fewer datasets included participant data for the other markers (appendix pp 22–26). This missingness of data for other markers was largely due to differences in study design and data collection rather than within-study participant non-response.

Meta-analysis of these studies showed a progressive reduction in sensitivity with increasing blood loss thresholds. For instance, a 200 mL threshold produced a summary sensitivity of 89·8% (95% CI 80·3–95·0), whereas sensitivity dropped to 47·9% (32·4–63·9) at a 1000 mL threshold (table 1). The DOR remained higher than 2 across all thresholds for measured blood loss, suggesting a consistently meaningful prognostic performance. At the conventional threshold of 500 mL or more, measured blood loss achieved a sensitivity of 75·7% (95% CI 60·3–86·4) and specificity of 81·4% (70·7–88·8). However, the predefined target for sensitivity (>80%) was achieved at a threshold of 300 mL or more (83·9% [95% CI 72·8–91·1]), but at the expense of a lower specificity of 54·8% (95% CI 38·0–70·5).

**Table 1:** Prognostic accuracy estimates per threshold for each clinical marker for the composite outcome of maternal death or severe morbidity

	**Sensitivity (95% CI)**	**Specificity (95% CI)**	**DOR (95% CI)**	**LR+ (95% CI)**	**LR– (95% CI)**
**Blood loss (n=305 523 women)**					
200 mL	89·8% (80·3–95·0)	30·9% (16·1–51·1)	4·0 (1·6–10·1)	1·3 (1·0–1·7)	0·3 (0·2–0·7)
250 mL	86·1% (76·4–92·2)	46·6% (30·7–63·1)	5·4 (3·0–9·7)	1·6 (1·2–2·1)	0·3 (0·2–0·5)
300 mL	83·9% (72·8–91·1)	54·8% (38·0–70·5)	6·3 (3·4–11·9)	1·9 (1·3–2·6)	0·3 (0·2–0·5)
350 mL	79·8% (68·6–87·7)	65·9% (51·0–78·2)	7·6 (4·4–13·3)	2·3 (1·6–3·3)	0·3 (0·2–0·5)
400 mL	78·9% (66·5–87·5)	71·2% (57·0–82·2)	9·3 (5·3–16·2)	2·7 (1·9–4·0)	0·3 (0·2–0·4)
450 mL	76·5% (63·1–86·1)	78·0% (66·4–86·4)	11·5 (6·7–20·0)	3·5 (2·4–5·1)	0·3 (0·2–0·5)
500 mL	75·7% (60·3–86·4)	81·4% (70·7–88·8)	13·6 (7·2–25·8)	4·1 (2·7–6·2)	0·3 (0·2–0·5)
550 mL	72·4% (55·3–84·7)	85·3% (76·6–91·2)	15·2 (7·4–31·3)	4·9 (3·2–7·7)	0·3 (0·2–0·5)
600 mL	70·9% (53·4–83·8)	87·3% (79·4–92·5)	16·8 (8·0–35·0)	5·6 (3·5–8·9)	0·3 (0·2–0·6)
700 mL	64·8% (47·3–79·0)	91·1% (85·1–94·9)	18·9 (9·6–37·2)	7·3 (4·6–11·7)	0·4 (0·2–0·6)
800 mL	58·9% (42·9–73·2)	93·7% (89·0–96·5)	21·4 (11·6–39·7)	9·4 (5·7–15·4)	0·4 (0·3–0·6)
1000 mL	47·9% (32·4–63·9)	97·1% (94·4–98·5)	30·5 (16·6–55·8)	16·3 (9·6–27·9)	0·5 (0·4–0·7)
**Systolic blood pressure (n=31 567 women)**					
90 mm Hg	14·0% (6·0–29·3)	97·3% (91·2–99·2)	5·9 (2·8–12·3)	5·2 (2·5–10·9)	0·9 (0·8–1·0)
100 mm Hg	31·7% (18·8–48·2)	85·9% (68·5–94·5)	2·8 (1·5–5·5)	2·2 (1·2–4·1)	0·8 (0·7–0·9)
110 mm Hg	55·9% (41·8–69·1)	54·5% (33·5–74·0)	1·5 (1·0–2·3)	1·2 (1·0–1·6)	0·8 (0·7–0·9)
120 mm Hg	74·2% (62·1–83·5)	23·9% (11·8–42·7)	0·9 (0·6–1·4)	1·0 (0·9–1·1)	1·1 (0·8–1·5)
130 mm Hg	85·2% (77·7–90·4)	7·5% (3·0–17·4)	0·5 (0·3–0·9)	0·9 (0·9–1·0)	2·0 (1·1–3·6)
140 mm Hg	94·1% (88·9–96·9)	2·0% (0·6–6·2)	0·3 (0·1–0·7)	1·0 (0·9–1·0)	3·0 (1·3–6·6)
**Diastolic blood pressure (n=31 567 women)**					
50 mm Hg	13·4% (5·1–30·8)	95·0% (85·2–98·4)	2·9 (1·3–6·3)	2·7 (1·3–5·5)	0·9 (0·8–1·0)
60 mm Hg	35·2% (21·0–52·8)	76·6% (53·7–90·3)	1·8 (1·0–3·1)	1·5 (1·0–2·4)	0·8 (0·7–1·0)
70 mm Hg	64·3% (48·3–77·7)	38·6% (22·5–57·7)	1·1 (0·7–1·8)	1·0 (0·9–1·3)	0·9 (0·7–1·2)
80 mm Hg	80·8% (70·6–88·1)	12·8% (6·7–22·9)	0·6 (0·4–0·9)	0·9 (0·9–1·0)	1·5 (1·1–2·1)
90 mm Hg	95·5% (89·6–98·1)	3·0% (1·5–5·7)	0·7 (0·4–1·2)	1·0 (1·0–1·0)	1·5 (0·8–2·7)
100 mm Hg	99·7% (82·8–100·0)	0·7% (0·3–1·4)	2·1 (0·0–104·4)	1·0 (1·0–1·0)	0·5 (0·0–22·6)
**Pulse rate (n=12 716 women)**					
60 bpm	97·5% (94·7–98·9)	0·6% (0·1–3·2)	0·2 (0·0–1·5)	1·0 (1·0–1·0)	4·1 (0·6–26·0)
70 bpm	93·9% (85·5–97·6)	8·5% (5·0–14·1)	1·4 (0·7–2·8)	1·0 (1·0–1·1)	0·7 (0·4–1·3)
80 bpm	88·2% (72·6–96·5)	27·3% (20·2–35·8)	2·8 (1·3–5·9)	1·2 (1·1–1·3)	0·4 (0·2–0·9)
90 bpm	75·0% (50·4–89·9)	54·8% (46·0–63·3)	3·6 (1·5–8·7)	1·7 (1·4–2·0)	0·5 (0·2–0·9)
100 bpm	58·2% (30·4–81·5)	77·4% (70·7–83·0)	4·8 (1·1–21·5)	2·6 (1·8–3·7)	0·5 (0·3–1·0)
110 bpm	36·9% (18·6–60·0)	89·9% (85·5–93·1)	5·2 (2·5–10·8)	3·6 (2·3–5·8)	0·7 (0·5–1·0)
**Shock index (n=12 716 women)**					
0·8	72·8% (59·0–83·3)	46·3% (32·6–60·6)	2·3 (1·3–4·2)	1·4 (1·1–1·7)	0·6 (0·4–0·9)
0·9	59·4% (41·6–75·0)	70·6% (56·1–81·8)	3·5 (1·7–7·3)	2·0 (1·3–3·1)	0·6 (0·4–0·9)
1·0	48·7% (27·6–70·3)	85·4% (74·6–92·1)	5·6 (2·3–13·8)	3·4 (1·9–6·1)	0·6 (0·4–0·9)
1·1	32·8% (16·6–54·4)	92·5% (85·7–96·2)	6·0 (2·5–14·5)	4·4 (2·2–8·8)	0·7 (0·6–1·0)
1·2	22·1% (9·3–44·2)	95·8% (91·3–98·0)	6·5 (2·8–15·1)	5·3 (2·6–10·6)	0·8 (0·7–1·0)
1·3	15·2% (4·3–41·3)	97·8% (94·9–99·0)	7·8 (2·8–21·5)	6·8 (2·9–16·0)	0·9 (0·7–1·1)

Bpm=beats per min. DOR=diagnostic odds ratio. LR+=positive likelihood ratio. LR–=negative likelihood ratio.

Systolic and diastolic blood pressure, pulse rate, and shock index had similar trade-offs between prognostic sensitivity and specificity, although not all thresholds examined had good predictive value (table 1). For systolic and diastolic blood pressure, the sensitivity decreased and specificity increased substantially with each 10 mm Hg decrement in threshold. DOR values were higher than 2 for systolic blood pressure of 100 mm Hg or lower and diastolic blood pressure of 50 mm Hg or lower, suggesting good discriminatory power at these thresholds. For pulse rate, sensitivity decreased and specificity increased sharply with every 10 bpm increment in threshold, although only thresholds above 80 bpm had a DOR greater than 2. Shock index showed a similar pattern to pulse rate, with sensitivity decreasing and specificity increasing considerably with each 0·1 unit increment in value; however, DOR remained greater than 2 for all thresholds, suggesting that it remained a useful predictor across the observed range of shock index scores. None of the clinical markers other than measured blood loss had any threshold that met the predefined target sensitivity exceeding 80% and specificity of at least 50% to justify their use as standalone clinical markers. The summary ROC curves showing the prognostic accuracy for each clinical marker for every available threshold are shown in the appendix (pp 28–33).

**Figure 2:**
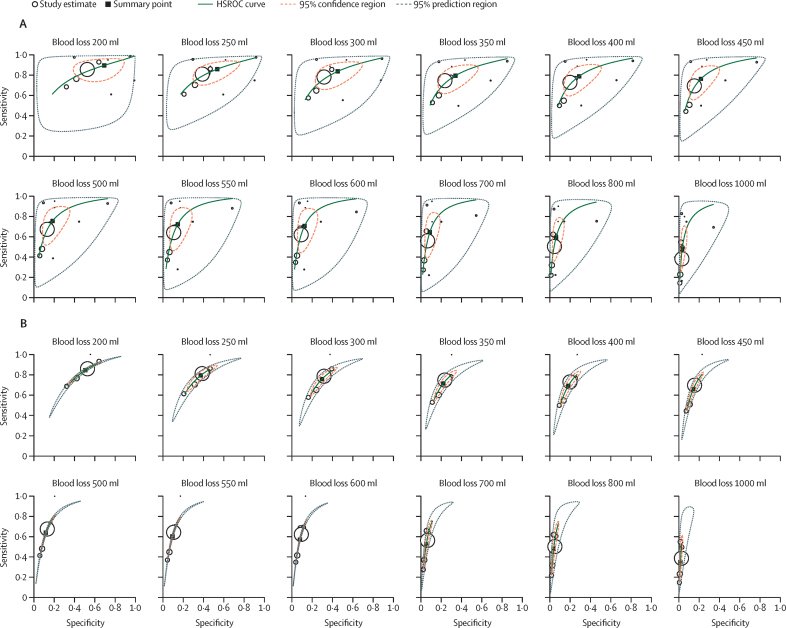
Summary ROC curves for blood loss thresholds showing measurement of blood loss as a source of heterogeneity The graphs show ROC curves for blood loss thresholds for the entire cohort (A) and for only the participants that had their blood loss weighed (B). HSROC=hierarchical summary receiver operating characteristic. ROC=receiver operating characteristic.

Visual inspection of the summary ROC plots for individual clinical markers shows moderate to substantial heterogeneity in prognostic accuracy across studies, as indicated by wide prediction regions around the summary points for sensitivity and specificity (appendix pp 28–33). Comparison of summary ROC plots for blood loss thresholds in women with weighed blood loss to plots for all women show much narrower prediction regions (figure 2), suggesting that the method of blood loss assessment could be a key source of between-study heterogeneity in prognostic accuracy estimates across studies.

Table 2 summarises the prognostic performance of the three decision rules for blood loss thresholds of less than 500 mL (≥300 mL to ≥450 mL). Across these blood loss thresholds, the first decision rule (any abnormal clinical marker) had high sensitivity (range 91·6–94·3%) but low specificity (29·3–40·9%); the second decision rule (blood loss and any other abnormal clinical marker) improved specificity (range 82·9–88·6%) but at the cost of sensitivity (63·4–64·3%); the third rule (a composite point scoring system) offered a more accurate and balanced performance for sensitivity (range 86·9–87·9%) and specificity (66·6–76·1%), with the highest DOR (14·4–21·1; table 2). As blood loss thresholds increased, the third rule had an increase in specificity without any substantial change in sensitivity. The summary ROC curves showing the prognostic accuracy for each decision rule by blood loss thresholds are shown in the appendix (pp 34–36). These plots also show wide prediction regions, suggesting variability in prognostic estimates across studies. However, the widths of the prediction regions are partly attributable to the fewer number of studies contributing to the pooled estimates.

**Table 2:** Prognostic accuracy estimates for combination of clinical markers by blood loss thresholds below 500 mL

	**Sensitivity (95% CI)**	**Specificity (95% CI)**	**DOR (95% CI)**	**LR+ (95% CI)**	**LR– (95% CI)**
**300 mL**					
Rule 1: any abnormal clinical marker (blood loss ≥300 mL, pulse rate >100 bpm, systolic blood pressure <100 mm Hg, diastolic blood pressure <60 mm Hg, or shock index >1)	94·3 (87·5–97·5)	29·3 (15·0–49·2)	6·8 (3·5–13·4)	1·3 (1·1–1·7)	0·2 (0·1–0·4)
Rule 2: blood loss plus any other abnormal clinical marker (blood loss ≥300 mL and pulse rate >100 bpm, systolic blood pressure <100 mm Hg, diastolic blood pressure <60 mm Hg, or shock index >1)	63·4 (37·7–79·2)	82·9 (61·5–93·6)	6·7 (3·4–12·9)	3·4 (1·8–6·3)	0·5 (0·3–0·8)
Rule 3: ≥2 points on a composite point scoring system (blood loss 300–499 mL is 1 point; pulse rate >100 bpm, systolic blood pressure <100 mm Hg, diastolic blood pressure <60 mm Hg, or shock index >1 is 1 point; and blood loss ≥500 mL is 2 points)	87·9 (76·1–94·3)	66·6 (st43·5–83·7)	14·4 (6·7–31·2)	2·6 (1·5–4·7)	0·2 (0·1–0·3)
**350 mL**					
Rule 1: any abnormal clinical marker (blood loss ≥350 mL, pulse rate >100 bpm, systolic blood pressure <100 mm Hg, diastolic blood pressure <60 mm Hg, or shock index >1)	91·6 (82·6–96·2)	34·1 (15·7–59·0)	5·7 (3·0–10·6)	1·4 (1·0–1·9)	0·2 (0·2–0·4)
Rule 2: blood loss plus any other abnormal clinical marker (blood loss ≥350 mL and pulse rate >100 bpm, systolic blood pressure <100 mm Hg, diastolic blood pressure <60 mm Hg, or shock index >1)	63·9 (36·6–84·4)	84·2 (63·4–94·3)	9·5 (4·1–21·8)	4·1 (2·0–8·3)	0·4 (0·2–0·8)
Rule 3: ≥2 points on a composite point scoring system (blood loss 350–499 mL is 1 point; pulse rate >100 bpm, systolic blood pressure <100 mm Hg, diastolic blood pressure <60 mm Hg, or shock index >1 is 1 point; and blood loss ≥500 mL is 2 points)	87·4 (75·4–94·0)	70·6 (48·5–86·0)	16·6 (7·0–39·6)	3·0 (1·6–5·5)	0·2 (0·1–0·3)
**400 mL**					
Rule 1: any abnormal clinical marker (blood loss ≥400 mL, pulse rate >100 bpm, systolic blood pressure <100 mm Hg, diastolic blood pressure <60 mm Hg, or shock index >1)	91·7 (82·8–96·2)	37·0 (19·0–59·6)	6·5 (3·6–11·8)	1·5 (1·1–1·9)	0·2 (0·1–0·4)
Rule 2: blood loss plus any other abnormal clinical marker (blood loss ≥400 mL and pulse rate >100 bpm, systolic blood pressure <100 mm Hg, diastolic blood pressure <60 mm Hg, or shock index >1)	64·2 (36·2–85·0)	86·2 (66·9–95·0)	11·1 (4·6–26·9)	4·6 (2·2–9·7)	0·4 (0·2–0·8)
Rule 3: ≥2 points on a composite point scoring system (blood loss 400–499 mL is 1 point; pulse rate >100 bpm, systolic blood pressure <100 mm Hg, diastolic blood pressure <60 mm Hg, or shock index >1 is 1 point; and blood loss ≥500 mL is 2 points)	87·4 (75·1–94·1)	73·1 (52·1–87·1)	18·8 (7·7–46·2)	3·2 (1·7–6·1)	0·2 (0·1–0·3)
**450 mL**					
Rule 1: any abnormal clinical marker (blood loss ≥450 mL, pulse rate >100 bpm, systolic blood pressure <100 mm Hg, diastolic blood pressure <60 mm Hg, or shock index >1)	91·6 (81·1–96·5)	40·9 (20·1–65·5)	7·5 (4·1–13·7)	1·5 (1·1–2·2)	0·2 (0·1–0·3)
Rule 2: blood loss plus any other abnormal clinical marker (blood loss ≥450 mL and pulse rate >100 bpm, systolic blood pressure <100 mm Hg, diastolic blood pressure <60 mm Hg, or shock index >1)	64·3 (35·2–85·7)	88·6 (71·3–96·0)	14·0 (5·4–36·4)	5·6 (2·6–12·4)	0·4 (0·2–0·8)
Rule 3: ≥2 points on a composite point scoring system (blood loss 450–499 mL is 1 point; pulse rate >100 bpm, systolic blood pressure <100 mm Hg, diastolic blood pressure <60 mm Hg, or shock index >1 is 1 point; and blood loss ≥500 mL is 2 points)	86·9 (74·2–93·9)	76·1 (56·7–88·5)	21·1 (8·0–55·3)	3·6 (1·9–6·9)	0·2 (0·1–0·3)

Decision rules 1–3 are based on analysis of 30 691 women. Bpm=beats per min. DOR=diagnostic odds ratio. LR+=positive likelihood ratio. LR–=negative likelihood ratio.

**Figure 3:**
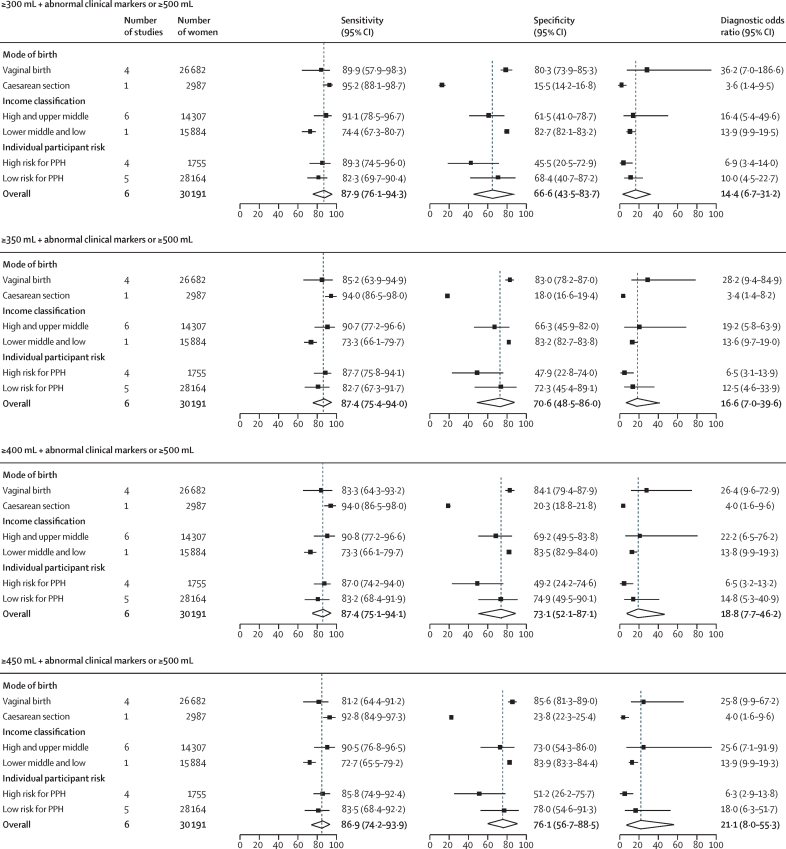
Subgroup accuracy estimates for a composite point scoring system combining blood loss between 300 and 499 mL with any other abnormal clinical marker and blood loss of 500 mL or more PPH=postpartum haemorrhage.

Subgroup estimates for measured blood loss thresholds starting from 300 mL (which achieved the target sensitivity) to the 1000 mL threshold were generated by mode of birth, country income level, and individual participant baseline risk (appendix pp 37–39). For mode of birth, subgroup estimates were based on meta-analyses of 11 studies for vaginal births, whereas estimates for caesarean births were drawn from a single study. Prognostic accuracy generally improved for all blood loss thresholds for the vaginal birth subgroups, whereas diagnostic performance of these blood loss thresholds in the caesarean subgroups was substantially lower, largely due to lower specificity (appendix pp 37–39). No substantial subgroup differences were observed by country income level or baseline risk across the blood loss thresholds examined (appendix pp 37–39). Subgroup estimates were also generated for the decision rule combining either blood loss of 300–499 mL plus any abnormal haemodynamic sign or blood loss of 500 mL or more by mode of birth, country income level, and individual participant baseline risk (figure 3). The prognostic estimates were much better for vaginal than caesarean births, although estimates for caesarean births were drawn from a single study, limiting the confidence in findings for this subgroup. However, no substantial difference was observed by country income level or baseline risk across the thresholds examined.

Sensitivity analyses restricting analyses to women who were treated at the conventional blood loss threshold and those with weighed blood loss did not find any substantial differences in prognostic estimates (appendix pp 40–43).

## Discussion

Our IPD meta-analysis of more than 300 000 women provides robust evidence that measured postpartum blood loss is an accurate clinical marker for predicting maternal death or severe morbidity. At the conventional threshold of 500 mL or more, measured blood loss achieved a sensitivity of 76% and specificity of 81%, indicating reasonable overall prognostic performance. However, a WHO global technical consultation underscored the importance of prioritising sensitivity in this context, with experts favouring earlier identification of women at risk over minimising false positives. In line with the preference of the WHO technical consultation experts, a threshold of 300 mL or more for measured blood loss met the desired sensitivity of more than 80%, but with reduced specificity (although this still met the prespecified sensitivity criterion of at least 50%). However, combining blood loss at the threshold of 300 mL or more with any abnormal haemodynamic markers or blood loss of 500 mL or more improved the specificity to 67%. In general, prognostic performance improved using combinations of blood loss thresholds lower than 500 mL and any abnormal haemodynamic sign or 500 mL or more of blood loss, with sensitivities ranging from 87% to 88% and specificities from 67% to 76%.

These findings suggest that although the conventional postpartum haemorrhage threshold of 500 mL or more remains a valid marker to identify women at risk of severe postpartum complications, it might not allow for timely intervention in all women. The evidence supports a lower threshold of blood loss (eg, ≥300 mL), particularly when combined with simple haemodynamic indicators, to improve early diagnosis of women at risk of serious postpartum complications. This approach is especially pertinent in settings with little access to comprehensive postpartum haemorrhage care, where early recognition and response are life-saving. Although use of a lower blood loss threshold for postpartum haemorrhage intervention could reduce specificity, potentially leading to unnecessary treatment in some women, this compromise can be offset by including abnormal haemodynamic signs before the conventional postpartum haemorrhage threshold of 500 mL is reached. Recognising these haemodynamic changes consistently across all health-care levels would remain a central challenge in optimising postpartum haemorrhage diagnosis and treatment in contexts where resources are scarce.

This study has several important strengths. This is the first IPD meta-analysis to date evaluating the diagnostic performance of clinical markers for postpartum haemorrhage, drawing on the largest postpartum haemorrhage datasets across diverse populations in the context of contemporary clinical practice. Previous efforts to develop prognostic models for postpartum haemorrhage have attempted to predict the risk of postpartum haemorrhage, not the risk of adverse outcomes related to postpartum haemorrhage.^25^, ^26^ The dataset assembled for this study is the largest of its kind and accounted for 84% of eligible individual participant data that were identified. Studies included were conducted in 23 countries, spanning all six WHO regions and four World Bank income classifications, ensuring robust global generalisability. An important strength of this study is that it is restricted to studies with measured blood loss, minimising measurement bias from visual estimations, which are known to underestimate blood loss.^27^ The included studies were mostly large, rigorous, randomised controlled trials with good quality data and very few losses to follow-up. The overall low risk of bias across key quality assessment domains increases confidence in the validity and reliability of our prognostic accuracy estimates. Furthermore, the data collected covered nearly all the potential tests and clinical markers that are predictive of death and complications, strengthening the validity of our findings. In addition, we used a rigorous scientific approach to data identification, harmonisation, and analysis.^28^, ^29^ We used a composite outcome that included death, severe complications, and life-saving interventions. This approach helps account for the varying availability of life-saving interventions. In regions where these interventions are less accessible, adverse outcomes might be more severe. For instance, in situations in which blood transfusion is not available when indicated, a woman could require surgical interventions to avoid further morbidity or death. Analysing these outcomes separately could introduce additional variability, making the results harder to interpret. To account for the varying prognostic performance of the markers assessed, we applied a point-based scoring system to optimise the balance between sensitivity and specificity, reflecting a pragmatic interpretation of prognostic relationships in clinical practice. Finally, we followed a robust consensus-building process to elicit the relative importance of prognostic accuracy measures in the context of postpartum haemorrhage, which is important for clinical interpretation and utility.

However, the study also has several limitations. First, not all eligible datasets were available for inclusion, the analysis included very few caesarean births, and some clinical markers were not reported in the largest studies included. As the blood loss patterns and baseline risks for caesarean births differ from those in vaginal births, our findings might not be generalisable to the caesarean population without additional validation. The scarce data on pulse rate, blood pressure, or shock index prevented robust multivariate assessments of all markers and reduced the overall power of analyses involving these markers. In addition, the relatively few total number of included studies restricted robust statistical assessment of heterogeneity and effect of covariates (eg, through meta-regression). Second, we used data collected from controlled research settings in which the quality of care and outcomes do not necessarily reflect the reality of routine care settings. For instance, the high proportion of uterotonic prophylaxis that is common in research settings might not be generalisable to rural or community settings, where this intervention is not the norm. Third, the data were not originally collected for the specific purpose of assessing the prognostic accuracy of clinical markers for postpartum haemorrhage-related morbidity and mortality. As a result, the timing of recording the clinical markers varied across the studies as they relate to the outcome and we were limited in our ability to conduct some key analyses of interest. For example, although most studies measured blood loss for up to 2 h after birth, they reported the final blood loss and not the blood loss at time of diagnosis or intervention to avert further blood loss. Likewise, the use of the most abnormal values before diagnosis of postpartum haemorrhage as predictors for markers measured at several timepoints creates potential circular reasoning, as the diagnosis could have been prompted by the abnormal marker value being evaluated as predictors. However, this issue is a limitation that is inherent in nearly all studies of early warning markers in acute obstetric care. Fourth, we did not have data on when first-response treatment was started and so we could not assess whether earlier intervention could have reduced total blood loss. Fifth, although treatment protocols were designed for all women diagnosed with postpartum haemorrhage in the included studies, variations in treatment regimens by women and by study could have influenced outcomes. The treatment paradox effect, in which treatment reduces both overall blood loss and the incidence of adverse outcomes related to postpartum haemorrhage, could have reduced the estimated predictive associations towards the null and it would have affected all clinical markers and decision rules. This treatment paradox effect implies that the prognostic accuracy of these markers was possibly underestimated, and caution is warranted in their interpretation. Despite these limitations, the large, diverse dataset and robust methodologies strengthen the validity and clinical relevance of our findings.

This study highlights several important avenues for future research. There is a need to validate these findings in real-world clinical and community settings and to assess the effect of implementing lower diagnostic thresholds, such as 300 mL or more of blood loss in combination with signs of abnormal haemodynamic changes, on both individual outcomes and broader health system performance and preparedness. Considering the resources required to conduct a large, sufficiently powered, test-and-treat interventional trial to assess the efficacy of alternative definitions of postpartum haemorrhage for clinical care, indirect evidence from existing studies might suffice in showing the effect of new diagnostic thresholds for postpartum haemorrhage. Nevertheless, implementation research will be essential to understand the practical barriers and enablers to adopting a more nuanced definition of postpartum haemorrhage, particularly in understaffed, low-income settings where monitoring is often scarce. Notably, our analyses for caesarean section were limited by the availability of a single study, which precluded meta-analysis and robust subgroup assessment; future research should focus specifically on generating prognostic accuracy data for caesarean births to address this gap. Further research should also explore how clinical markers might be integrated with emerging technologies, such as wearable monitoring systems or point-of-care diagnostics that could signal when the criteria are met, to enable earlier and more accurate identification of women at risk of death or severe morbidity from postpartum haemorrhage. Importantly, such work would support the evolving role of precision public health in maternal care and would allow tailored definitions of postpartum haemorrhage by incorporating individual-level characteristics to more accurately determine clinically significant blood loss.

Our findings challenge the exclusive use of the conventional threshold of 500 mL or more of blood loss for diagnosing postpartum haemorrhage and initiating treatment, particularly in low-resource settings, where delays are more likely to result in deaths. Additionally adopting a lower blood loss threshold in combination with abnormal haemodynamic signs could enable better preparedness, earlier recognition of women at risk of clinical deterioration, and potentially improve maternal outcomes. However, this could have substantial resource implications for health systems, including increased demands on staff training and care organisation, particularly in settings where routine monitoring of vital signs and objective blood loss measurement are not yet standard practice. On the contrary, as the key vital signs that were shown to be prognostically valuable (pulse, blood pressure, and shock index) are already core components of maternal early warning systems (eg, the Modified Early Obstetric Warning Score), integrating our findings for timelier and more effective management of women at risk of life-threatening postpartum haemorrhage is likely to be less resource-intensive in settings where such systems are already in place. It is important to note that the use of these diagnostic criteria could lead to an increase in the number of postpartum haemorrhage diagnoses, especially in settings that are simultaneously introducing objective assessment of blood loss and for caesarean births, for which the blood loss is generally greater than vaginal births.^15^ It is essential that policy makers and facility leaders do not misinterpret this increase for a decline in the quality of maternal care. In fact, the opposite is likely to be true. With prompt application of the WHO-recommended postpartum haemorrhage first-response treatment bundle,^27^ the incidence of more life-threatening blood loss (eg, ≥1000 mL) is expected to substantially decrease.

Although increased diagnoses of postpartum haemorrhage could lead to a higher demand for commodities and supplies necessary for first-response treatment, in many settings, this additional cost is likely to be offset by a reduction in the need for more invasive, resource-intensive treatments for severe postpartum haemorrhage complications, such as blood transfusions and admissions to intensive care units. Ultimately, the implementation of a nuanced definition of postpartum haemorrhage is anticipated to increase early detection of postpartum haemorrhage, improve quality of care, and reduce morbidity and mortality related to postpartum haemorrhage globally.

### WHO Consortium on Postpartum Haemorrhage Definition

### Contributors

### Data sharing

The individual participant data (IPD) analysed in this Article were shared with the WHO exclusively for the purpose of conducting this IPD meta-analysis to reappraise the definition of postpartum haemorrhage. Researchers interested in accessing data from the original studies should contact the corresponding authors of those studies directly. Access will be subject to the approval of data owners and applicable data sharing agreements.

## Declaration of interests

FA, AC, AD, AMG, OTO, and IY were coauthors for E-MOTIVE trial, the largest included trial. RCP received a direct payment from Organon for giving a lecture on postpartum haemorrhage management. LS has received consulting fees and payment or honoraria from Ferring Pharmaceuticals, Bayer, GlaxoSmithKline, Pfizer, Organon, and Norgine. All other authors declare no competing interests.

## References

[1] WHO (2025).

[2] Calvert C, John J, Nzvere FP (2021). Maternal mortality in the Covid-19 pandemic: findings from a rapid systematic review. Glob Health Action.

[3] Cresswell JA, Alexander M, Chong MYC (2025). Global and regional causes of maternal deaths 2009–20: a WHO systematic analysis. Lancet Glob Health.

[4] El-Refaey H, Rodeck C (2003). Post-partum haemorrhage: definitions, medical and surgical management. A time for change. Br Med Bull.

[5] Davidson M, Junkin R, Clark A (2023). P24 Impact of focusing definitions of postpartum haemorrhage. Int J Obstet Anesth.

[6] de Vries PLM, Deneux-Tharaux C, Baud D (2023). Postpartum haemorrhage in high-resource settings: variations in clinical management and future research directions based on a comparative study of national guidelines. BJOG.

[7] Souza JP, Day LT, Rezende-Gomes AC (2024). A global analysis of the determinants of maternal health and transitions in maternal mortality. Lancet Glob Health.

[8] Debray TPA, Moons KGM, van Valkenhoef G (2015). Get real in individual participant data (IPD) meta-analysis: a review of the methodology. Res Synth Methods.

[9] Weeks J, Cuthbert A, Alfirevic Z (2023). Trustworthiness assessment as an inclusion criterion for systematic reviews—what is the impact on results?. Cochrane Ev Synth.

[10] Lee J, Mulder F, Leeflang M, Wolff R, Whiting P, Bossuyt PM (2022). QUAPAS: an adaptation of the QUADAS-2 tool to assess prognostic accuracy studies. Ann Intern Med.

[11] Macaskill P, Takwoingi Y, Deeks JJ, Gatsonis C, Deeks JJ, Bossuyt PM, Leeflang MM, Takwoingi Y (2023). Cochrane handbook for systematic reviews of diagnostic test accuracy.

[12] Naaktgeboren CA, Bertens LCM, van Smeden M, de Groot JA, Moons KGM, Reitsma JB (2013). Value of composite reference standards in diagnostic research. BMJ.

[13] Devall A, Nausheen S, Muhammad S (2025). Early detection and bundled treatment of postpartum haemorrhage with the E-MOTIVE intervention: a prospective pre-post intervention study in Pakistan. Lancet Obstet Gynaecol Womens Health.

[14] Durocher J, Dzuba IG, Carroli G (2019). Does route matter? Impact of route of oxytocin administration on postpartum bleeding: a double-blind, randomized controlled trial. PLoS One.

[15] Gallos I, Devall A, Martin J (2023). Randomized trial of early detection and treatment of postpartum hemorrhage. N Engl J Med.

[16] Gülmezoglu AM, Villar J, Ngoc NT (2001). WHO multicentre randomised trial of misoprostol in the management of the third stage of labour. Lancet.

[17] Gülmezoglu AM, Lumbiganon P, Landoulsi S (2012). Active management of the third stage of labour with and without controlled cord traction: a randomised, controlled, non-inferiority trial. Lancet.

[18] Mammoliti K-M, Martin J, Devall A, et al. When are postpartum haemorrhages detected? A nested observational study within the E-MOTIVE cluster-randomised trial. *Lancet Glob Health* (in press). 10.1016/S2214-109X(25)00302-X PMC12535819 41109265

[19] Mobeen N, Durocher J, Zuberi N (2011). Administration of misoprostol by trained traditional birth attendants to prevent postpartum haemorrhage in homebirths in Pakistan: a randomised placebo-controlled trial. BJOG.

[20] Pacagnella RC, Borovac-Pinheiro A, Silveira C (2022). The golden hour for postpartum hemorrhage: results from a prospective cohort study. Int J Gynecol Obstet.

[21] Haslinger C, Korte W, Hothorn T, Brun R, Greenberg C, Zimmermann R (2020). The impact of prepartum factor XIII activity on postpartum blood loss. J Thromb Haemost.

[22] Sentilhes L, Winer N, Azria E (2018). Tranexamic acid for the prevention of blood loss after vaginal delivery. N Engl J Med.

[23] Sentilhes L, Sénat MV, Le Lous M (2021). Tranexamic acid for the prevention of blood loss after cesarean delivery. N Engl J Med.

[24] Widmer M, Piaggio G, Nguyen TMH (2018). Heat-stable carbetocin versus oxytocin to prevent hemorrhage after vaginal birth. N Engl J Med.

[25] Carr BL, Jahangirifar M, Nicholson AE, Li W, Mol BW, Licqurish S (2022). Predicting postpartum haemorrhage: a systematic review of prognostic models. Aust N Z J Obstet Gynaecol.

[26] Neary C, Naheed S, McLernon DJ, Black M (2021). Predicting risk of postpartum haemorrhage: a systematic review. BJOG.

[27] WHO (2023).

[28] Knottnerus JA, Buntinx F (2009).

[29] Riley RD, van der Windt D, Croft P, Moons KGM (2019). Prognosis research in healthcare: concepts, methods, and impact.

