# Supplementary material for: Prognostic accuracy of clinical markers of postpartum bleeding in predicting maternal mortality or severe morbidity: a WHO individual participant data meta-analysis
Source: Lancet. 2025 Oct 25;406(10514):1969–82. doi: 10.1016/S0140-6736(25)01639-3 (PMC12549477; doi:10.1016/S0140-6736(25)01639-3)
Supplement: Supplementary appendix [file mmc1.pdf]

THE LANCET

Supplementary appendix

This appendix formed part of the original submission and has been peer reviewed. We post it as supplied by the authors.

Supplement to: Gallos I, Williams CR, Price MJ, et al. Prognostic accuracy of clinical markers of postpartum bleeding in predicting maternal mortality or severe morbidity: a WHO individual participant data meta-analysis. *Lancet* 2025; published online Oct 4. https://doi.org/10.1016/S0140-6736(25)01639-3.

Supplementary Appendix

Prognostic accuracy of clinical markers of postpartum bleeding in predicting maternal mortality or severe morbidity

Ioannis Gallos MD, Caitlin R Williams PhD, Malcolm J Price PhD, Aurelio Tobias PhD, Prof Adam Devall PhD, Prof John Allotey PhD, Fernando Althabe MSc, Jenny A Cresswell PhD, Jill Durocher BA, A Metin Gülmezoglu PhD, Prof Christian Haslinger MD, Prof Rodolfo C Pacagnella MD, Loïc Sentilhes MD, Soha Sobhy MD, Idnan Yunas MBBChir, Prof Jonathan J Deeks PhD, Prof Arri Coomarasamy MD, Olufemi T Oladapo MD *for the WHO Consortium on Postpartum Haemorrhage Definition*

Table of Contents

1. Overview of the WHO Postpartum Haemorrhage Definition Project 3

Background and Rationale 3

Objectives of the Project 3

Aim of the Individual Participant Data Meta-analysis 3

2. Methods for Individual Participant Data Meta-analysis 4

Protocol Registration and Reporting Standards 4

Search Strategy and Study Selection 4

Individual Participant Data Request, Collection, and Security 5

Data Harmonisation, Standardisation, and Integrity Checking 6

Harmonization procedures 6

Standardization procedures 6

Integrity Checks and Trustworthiness Assessment 6

Clinical Outcome Definitions 8

Prognostic Outcome Definitions 8

Risk of Bias and Applicability Assessment (QUAPAS) 9

Statistical Analysis Plan 9

Descriptive statistics 9

Assessment of Heterogeneity 10

Sensitivity Analyses 10

Interpretation of Results 11

Deviations from the protocol 11

3. Eligibility Criteria Checklist 11

4. Data Sharing Standards (DSS) 14

Data Structure 14

Data Dictionary and Anonymization 14

Data Sharing and Security Procedures 18

5. Supplementary Results 22

Table S1: Included Studies in IPD Meta-Analysis 22

Table S2: Summary of Clinical Characteristics of Participants 26

Figure S1. Risk of Bias Assessment. 27

Figures S2-S6. Summary ROC Plots for Individual Markers 28

Figures S7-S9. Summary ROC Plots for Decision Rules 34

Figure S10. Subgroup analyses (by mode of birth, income, risk). 37

Figures S11. Sensitivity Analyses (by treatment threshold) 40

Tables S3–S4: Prognostic Accuracy Estimates (for sensitivity analyses) 42

6. Supplementary Materials 44

PRISMA-IPD Checklist 44

Consortium Members and Collaborators 48

7. References 50

1. Overview of the WHO Postpartum Haemorrhage Definition Project

Background and Rationale

Postpartum haemorrhage (PPH) remains the leading cause of maternal mortality globally. Beyond mortality, PPH contributes to significant long-term morbidity, including reproductive disability, psychological trauma, and chronic health conditions. Despite its critical impact, there is no universally accepted definition of PPH. The most widely used definition, blood loss of 500 mL or more following childbirth, originated from a 1962 observational study of 75 women in the United States.[1] To date, the definition of PPH lacks a robust evidence base.

A 2023 systematic review of international guidelines revealed substantial variation in PPH definitions, with discrepancies in blood loss thresholds, timing of measurement, and inclusion of clinical signs of haemodynamic instability.[2] This inconsistency extends to clinical trials, where over ten different blood loss thresholds have been used, complicating efforts to synthesize evidence and guide practice.[3]

The rationale for reappraising the definition of PPH stems from the urgent need to improve maternal outcomes by addressing the limitations of the current definition, which is based on outdated, non-evidence-based thresholds and fails to reflect contemporary clinical practices, population health changes, and risk profiles. Substantial variation in how PPH is defined across guidelines and research studies has led to confusion, delayed interventions, and inconsistent data, undermining clinical care, epidemiological surveillance, and health system planning. Emerging evidence suggests that earlier intervention at lower blood loss thresholds can significantly reduce severe PPH and other complications, highlighting the inadequacy of the current benchmark. A revised, evidence-based, and context-sensitive definition is essential to guide timely clinical action, standardise research, improve data comparability, and support health system functions such as training, coding, and policy-making, with the ultimate goal of ensuring better care and outcomes for women globally.

Objectives of the Project

The primary objective of this project is to reappraise the current WHO definition of PPH with the aim of refining, revising, or updating it for contemporary clinical practice, research, and policy. To achieve this objective, WHO aims to develop and apply an evidence-based framework that includes rigorous review of research evidence underpinning consensus-based, distribution-based, prognosis-based, and therapy-based approaches.

This document presents the detailed methodology and supplemental results for the prognostic accuracy research, an individual participant data (IPD) meta-analysis, which underpins the prognosis-based approach to PPH definition.

Aim of the Individual Participant Data Meta-analysis

The aim of the study is to generate robust estimates of prognostic accuracy of commonly used clinical markers of postpartum bleeding including measured blood loss, pulse rate, systolic and diastolic blood pressure, and shock index in predicting maternal mortality or severe morbidity.

2. Methods for Individual Participant Data Meta-analysis

Protocol Registration and Reporting Standards

We conducted an individual participant data (IPD) meta-analysis in accordance with a prespecified protocol, registered on PROSPERO (CRD420251034918), and available via the WHO website. This meta-analysis is reported in line with the PRISMA-IPD guidelines; a completed checklist is provided in Page 43.

Search Strategy and Study Selection

Eligible studies were identified via a global call for data issued by WHO and disseminated using all feasible WHO channels. The aim of the call was to obtain published and unpublished datasets containing data on prognostic clinical markers of postpartum bleeding and outcomes.

In addition to the global call for data, we conducted systematic searches of PubMed, MEDLINE, Embase, the Cochrane Library, and the WHO International Clinical Trials Registry Platform (ICTRP) up to 06 November 2024. Search terms included combinations of "pregnancy", "birth", "postpartum haemorrhage", "blood loss", "pulse", "blood pressure", "shock index", "haemoglobin", and "lactate" (see full strategy below). There were no restrictions on language or publication status. Reference lists of eligible studies and reviews were screened, and conference proceedings were searched for additional unpublished data.

Embase <1974 to 2024 November 06>

Ovid MEDLINE(R) ALL <1946 to November 06, 2024>

1	(Shock index or blood pressure or heart rate or pulse level or blood loss or lactate or fibrinogen or prothrombin or Christmas factor or Stuart-Prower factor or plasma thromboplastin or Hageman factor).ab,ti.	1 778 346
2	(pph or postpartum hemorrhag* or postpartum haemorrhag* or post partum hemorrhag* or post partum haemorrhag*).ti. or (pph or postpartum hemorrhag* or postpartum haemorrhag* or post partum hemorrhag* or post partum haemorrhag*).ab.	32 887
3	(uterotonics or blood transfusion or balloon tamponade or laparotomy or hysterectomy or maternal death or blood loss or mortality or near miss or ICU admission or surgical procedure or uterine artery ligation or b-lynch suture).ab,ti.	3 215 785
4	(severe and ((obstetric or postpartum) adj5 h?emorrhage*)).ab,ti.	5 476
5	3 or 4	3 217 705
6	1 and 2 and 5	5 418
7	limit 6 to English language	5 144
8	limit 7 to full text	1 384
9	limit 8 to human	1 317
10	limit 9 to humans	1 317
11	limit 10 to "remove preprint records"	1 317

Two authors (JA, SS) independently screened titles, abstracts, and full texts for eligibility; discrepancies were resolved by consensus. The PRISMA-IPD flow diagram (Figure 1, main manuscript) documents study identification, screening, inclusion, and exclusion.

The study eligibility criteria included the following:

1. Cohort design from observational or experimental studies.
2. Study size of at least 200 participants.
3. Data available on objectively measured blood loss by weighing blood loss (ideal) or via tools for fixed volumetric assessment such as calibrated drapes under participant's buttocks or measuring jars (acceptable), pulse rate, respiratory rate, blood pressure, abnormal uterine tone, or any clinical signs and symptoms of haemodynamic instability [full list in Figure 1]. Studies that visually estimated blood loss are not eligible because of the inaccuracy of this method.
4. Data available on at least one clinical outcome (e.g., blood transfusion, surgical interventions, morbidity, or mortality) [Figure 1].
5. Evidence of ethics committee approval before data collection.
6. Data collected using a standardised protocol and/or through clinical/public health care encounter after 1990.
7. Data holder can sign a legal data sharing agreement with WHO.
8. Study not conducted exclusively on women diagnosed with postpartum haemorrhage.

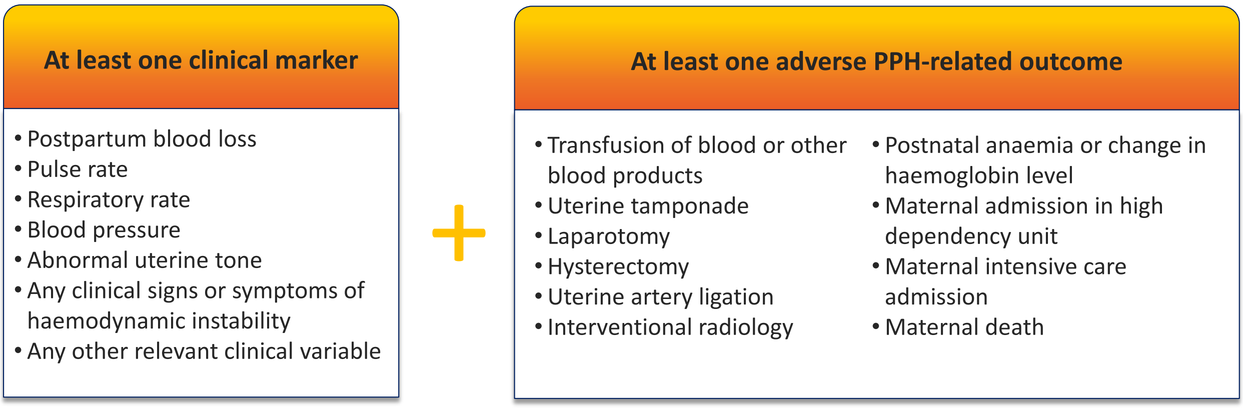

Eligibility criteria for clinical markers and outcome variables.

Individual Participant Data Request, Collection, and Security

The global call for data included an online eligibility form to obtain information on the study eligibility criteria and principal investigators (PIs). For studies that appeared to meet the eligibility criteria, WHO contacted the PIs and initiated the process of obtaining the datasets. Authors of eligible studies were invited by email (up to three reminders) to contribute de-identified individual participant data via a secure WHO data portal. For records of studies that appeared eligible through the literature searches, PIs were contacted and asked to confirm eligibility through an online checklist. All potentially eligible data were sought irrespective of the data that was included in the published reports. For PIs who expressed interest directly through the WHO global call for data, they were also asked to complete the online checklist and follow a similar process for confirming eligibility (see Page 11 for the eligibility checklist). Two authors (IG, CRW) reviewed the submitted data and contacted the PIs for any queries regarding the eligibility of the data. Study selection was conducted independently by the two authors, and any disagreements were resolved by discussion and consensus.

After initial confirmation of eligibility through the checklist and in consultation with the PIs, IPD were requested from eligible records, and standard data sharing agreements were prepared between WHO and institutions that owned the data. Data sharing agreements were executed between WHO and each participating institution, covering permissible data uses, access, and publication rights. Each PI or their

corresponding institution was asked to supply raw datasets, accompanying data dictionaries, original study protocols, and documentation of measurement methods. All IPD were stored on encrypted, access-restricted server at WHO headquarters, with audit trails for data access and modifications. All shared datasets were cross-referenced against published protocols and study reports. The management and updates of the repository was the sole and exclusive responsibility of WHO staff and PIs collaborating with WHO had access only to their own data. For the records for which IPD were not provided, the possibility of using aggregate data for the purpose of this analysis was explored but the data required for this analysis were not available in the published reports.

Data Harmonisation, Standardisation, and Integrity Checking

Harmonization procedures

The PIs were provided with clear instructions about the required variables to share, the format, and the variable definitions as per publicly available data sharing standards (Page 14). Upon receipt of the data by WHO, the data variables in each dataset were mapped to a common variable dictionary, and checked for accuracy, validity, and internal consistency against the study protocols and published and unpublished reports. Specific checks included inconsistencies in recruitment dates, eligibility criteria, sample sizes, and outcome reporting. Common discrepancies included mismatched enrollment periods, inclusion of ineligible participants (e.g., without available outcomes), and implausible or illogical data entries such as outcome dates preceding randomization. Consistency checks per dataset were carried out through descriptive summary statistics and distribution graph analyses for each variable and by each outcome. Discrepancies in definitions, coding, or units (e.g., blood loss, blood pressure, pulse rate) were resolved through direct consultation with the PIs. If the PIs did not respond to queries, or their responses were unsatisfactory to address the issue(s), the WHO Secretariat determined whether to set a particular value to missing or to exclude a dataset until further information became available. For the harmonized dataset, one author (MJP) recoded and transformed data as required for consistency across datasets according to publicly available data sharing standards issued with the global call. Mapping of variables and checks of all the files for each dataset were verified by at least two authors (CRW, IG).

Standardization procedures

Data with different measurement units were converted according to the international system of units. Derived values were computed when their components were measured contemporaneously (e.g., shock index from pulse and blood pressure). For clinical markers measured at several time points (e.g., blood loss, pulse and blood pressure), we extracted measurements at 15, 30, 45, and 60 minutes after birth. For the primary analysis, we used the most abnormal value before the diagnosis of PPH was made. For measured blood loss, we extracted the total blood loss following birth at the time of diagnosis, if available, and the total blood loss when measurement was stopped. Any additional blood loss from visual estimation was not used. We extracted the outcomes of interest occurring after birth and until hospital discharge, irrespective of whether these were caused by excessive blood loss.

Integrity Checks and Trustworthiness Assessment

All datasets underwent systematic integrity assessment checks adapted from the Cochrane Pregnancy and Childbirth Trustworthiness Screening Tool.[1] If any issues were identified, the PIs were contacted for further information. If the PIs were not contactable or did not provide sufficient clarification to

address the issue(s), we would have excluded these studies from analyses until further information became available. However, we did not identify any integrity issues with any of the shared datasets.

Integrity checks descriptions adapted from the Cochrane Pregnancy and Childbirth Trustworthiness Screening Tool.[1]

Evaluation Criteria	How It Was Assessed
**Study Governance**	
Retraction or concern notices	Reviewed the Retraction Watch database for any retraction notices or expressions of concern related to the study.
Prospective registration	Verified whether the study was registered before participant enrollment by comparing registration and enrollment dates.
Ethics approval	Searched for documented ethics approval in trial registries, publications, or by contacting PIs.
Availability of protocol	Checked trial registries, publication databases, and contacted investigators to confirm the existence of a study protocol.
Responsiveness of PIs	Attempted contact of PIs and reported responsiveness.
Provision of IPD	Determined whether IPD were shared upon request and whether reasons for non-provision were justified.
**Baseline Characteristics**	
Similarity of participant data	Examined distributions of baseline characteristics for signs of implausible uniformity, such as identical or narrowly distributed values.
**Feasibility Indicators**	
Plausibility of study characteristics	Assessed whether recruitment rates, composite outcome incidence, and other reported features were realistic given the study context.
Follow-up completeness	Evaluated whether minimal or no loss to follow-up was plausible based on study duration and population.
**Outcome Reporting**	
Plausibility of results	Reviewed effect sizes and outcome distributions for signs of implausibility, such as unexpectedly large treatment effects, negative values for blood loss.
**Additional IPD Checks**	
Duplicate entries	Inspected datasets for repeated or copy-pasted baseline data entries without justification.
Digit preference	Analyzed numerical data with detailed descriptive statistics and histograms for non-random patterns in terminal digits, which may indicate rounding or fabrication.
Baseline imbalances	Identified any major differences in baseline characteristics between groups, particularly for key prognostic variables.
Similarity of outcomes	Assessed whether outcome distributions across groups were implausibly similar, suggesting potential data issues.

Evaluation Criteria	How It Was Assessed
Protocol date consistency	Verified that reported dates (e.g., death, enrollment) followed a logical sequence and did not precede study initiation.

Clinical Outcome Definitions

Clinical outcomes of interest included all-cause maternal mortality, transfusion of whole blood and/or blood products, mechanical or surgical intervention to stop bleeding [uterine tamponade, laparotomy, vessel ligation, compression sutures, interventional radiology, hysterectomy], maternal sepsis, organ system failure, high-dependency and intensive care admission. The primary outcome was a composite of all-cause maternal death or life-threatening morbidity (transfusion, major surgery, ICU/high-dependency admission). The definitions of each of the components of the composite outcome are presented below.

Component	Description of individual components
**Transfusion**	Transfusion of red cells, whole blood, or any other blood products during the postpartum period
**Major Surgery**	Surgical procedures to control postpartum bleeding and other complications including uterine tamponade, laparotomy with uterine artery or internal iliac artery ligation, uterine compression sutures, or interventional radiology, or hysterectomy.
**Morbidity**	Postpartum morbidity including maternal sepsis, admission to high dependency or intensive care, organ failure, or transfer to higher level of care.
**Death**	Any maternal death during the postpartum period from any cause.

Prognostic Outcome Definitions

The statistical outcomes of interest included measures of prognostic accuracy, including sensitivity, specificity, diagnostic odds ratio (DOR), and positive and negative likelihood ratios by selected thresholds for each clinical marker.

For the purpose of this analysis, sensitivity was defined as the proportion of women who experienced the composite outcome (maternal death or severe morbidity) and were correctly identified as high risk by the clinical marker (true positives). In other words, it is the proportion of true positive cases among all women who had the composite outcome. Specificity was defined as the proportion of women who did not experience the composite outcome and were correctly identified as not being at high risk by the clinical marker, i.e., the proportion of true negatives among all women who did not have the composite outcome.

DOR was defined as the ratio of the odds of a positive test result in women with the composite outcome to the odds of a positive test result in women without the composite outcome, providing a single indicator of test performance that combines sensitivity and specificity.

Positive Likelihood Ratio (LR+) was defined as the ratio of the probability of a positive test result in women with the composite outcome to the probability of a positive test result in women without the composite outcome, indicating how much the odds of the composite outcome increase when the test is positive. Negative Likelihood Ratio (LR−) was defined as the ratio of the probability of a negative test

result in women with the composite outcome to the probability of a negative test result in women without the composite outcome, indicating how much the odds of the outcome decrease when the test is negative.

Risk of Bias and Applicability Assessment (QUAPAS)

Risk of bias and applicability in the individual studies was assessed independently by at least two reviewers (CRW, IG, JA) using the Quality Assessment of Prognostic Accuracy (QUAPAS) tool.[2] Reviewers did not assess risk of bias for studies they were directly involved. The QUAPAS tool assessed the risk of bias and concerns about applicability of studies across five domains: participants, index test, outcome, flow and timing, and analysis. We considered all enrolled participants to be included in the analysis if data were available for >95% of all participants on the clinical markers and reported outcomes of interest, regardless of whether the data were included in the analysis for the primary paper. Disagreements were resolved through discussion and consensus.

Statistical Analysis Plan

The statistical analysis closely followed established best practices for IPD-based diagnostic accuracy studies, including PRISMA-IPD guidance[3] and recommendations from the Cochrane Prognosis Methods Group, with specific refinements and methodological clarifications incorporated for clarity and completeness.

The primary objective of the analysis was to estimate and compare the prognostic accuracy (sensitivity, specificity, diagnostic odds ratio) of clinical markers of PPH in predicting a composite outcome of maternal death or severe morbidity (i.e., transfusion, surgery, ICU admission). The secondary objectives were to (i) identify optimal thresholds for each marker and combinations thereof; (ii) evaluate performance across subgroups by mode of birth, country income level, and baseline PPH risk; and (iii) to explore sensitivity and robustness of findings.

Descriptive statistics

Baseline characteristics (age, parity, haemoglobin, mode of birth, etc.) across all included studies were presented using medians (with interquartile ranges) for continuous data and proportions for dichotomous data. Incidence of the composite outcome and its components (maternal death, blood transfusion, mechanical or surgical interventions, or intensive care admissions) were presented as proportions.

We used a two-stage approach to generate prognostic sensitivities, specificities, DOR, positive and negative likelihood ratios and their confidence intervals for different thresholds of each clinical marker.

- Stage 1: Within each included study, we constructed 2×2 contingency tables at each pre-specified threshold for each clinical marker. From these tables, we calculated sensitivity, specificity, positive and negative likelihood ratios (LR+ and LR−), and the diagnostic odds ratio (DOR). Confidence intervals were calculated using binomial exact method.
- Stage 2: The individual study estimates were synthesized using a bivariate random-effects meta-analysis model, which jointly modeled logit-transformed sensitivity and specificity, accounting for the correlation between them and between-study variability.[4] This model yielded pooled estimates of sensitivity and specificity, a hierarchical summary receiver operating characteristic (HSROC) curve, a summary point, and 95% confidence and

prediction regions. Heterogeneity is to be expected in results of test accuracy studies, thus random-effects models are required to describe the variability in test accuracy across studies.[5]

We presented results in tables and graphically with circles showing the individual study estimates, summary curve from the hierarchical summary receiver operating characteristic, a summary operating point (i.e., summary values for sensitivity and specificity), 95% confidence region for the summary operating point, and 95% prediction region (confidence region for a forecast of the true sensitivity and specificity in a future study). A clinical marker was considered predictive if the diagnostic odds ratio for at least one of its thresholds is >2.

We calculated the prognostic accuracy measures at 50 mL incremental thresholds (above the median) for blood loss, at 10 beats per minute for pulse rate, 10 mmHg for systolic and diastolic blood pressure, and 0·1-unit for shock index. A clinical marker was considered predictive if the DOR suggested it was informative and >2 for at least one threshold. We also examined prognostic accuracy of three pre-defined decision rules combining clinical markers, to investigate whether combinations of markers improved accuracy over any individual marker alone. These rules were developed a priori based on clinical rationale, expert consensus, and literature review, following recommended principles for constructing composite rules to evaluate combinations of imperfect tests in diagnostic research. In the first decision rule, women were classified to be at high risk of death or severe morbidity if any one marker were abnormal (e.g., blood loss above target abnormal threshold, pulse >100 beats per minute for tachycardia, systolic blood pressure <100 mmHg or diastolic blood pressure <60 mmHg for hypotension, or shock index >1 for shock). In the second decision rule, women were classified as high risk of death or severe morbidity if blood loss and at least one other clinical marker were abnormal. The third rule was a composite scoring system that assigned 1 point for blood loss thresholds prioritizing early detection of women at high risk (i.e., high sensitivity), a maximum of 1 point for any other abnormal marker, and 2 points for blood loss thresholds demonstrating strong predictive power to identify women at high risk (i.e., high specificity and DOR). Women were classified as high risk if their total score was 2 or more.

Assessment of Heterogeneity

Heterogeneity was assessed through subgroup analyses conducted using the bivariate model by mode of birth (vaginal versus caesarean), World Bank classification by country income level for 2024-2025 (high-income versus low- and middle-income countries), and individual baseline risk (high versus low), where sufficient data for the subgroups were available. Participants were defined as high risk if they have any of the known risk factors (e.g., anaemia, grand-multiparity (5 or more births), previous PPH, previous caesarean birth, pre-eclampsia, BMI ≥ 30, induction of labour, episiotomy, retained placenta, birthweight ≥ 4500 g, multiple pregnancy, assisted vaginal birth or caesarean birth). Heterogeneity was visually inspected using ROC plots and quantified via confidence intervals of the DORs from bivariate models.

Sensitivity Analyses

We explored the robustness of the findings through sensitivity analyses conducted by removing populations with different prognoses (e.g., participants who had volumetric assessment of blood loss) or participants who had different test thresholds for initiating treatment (e.g., diagnosing and treating women with blood loss of 300 mL or more instead of the conventional diagnostic threshold of 500 mL or more). Subgroup and sensitivity results were examined by comparing the differences in DORs and prediction graphs in summary ROC plots.

All meta-analyses were conducted using Stata 17 (StataCorp, College Station, TX, 2021) and the metandi[4] command to derive summary statistics per clinical marker and perform meta-analysis of prognostic accuracy studies.

Interpretation of Results

WHO convened a technical consultation of international group of experts (n=26) from all WHO regions to review the preliminary results and decide on the relative value they place on the sensitivity and specificity of predictive clinical markers for the composite outcome of interest. The preferred trade-offs between sensitivity (>80%) and specificity (at least 50%) was achieved through consensus.

Deviations from the protocol

1. In the published protocol it was planned to include postnatal anaemia in the composite outcome. The harmonized dataset contained data on postpartum haemoglobin level from 8 datasets and 12 991 women. However, there were concerns about this outcome as in many studies women with severe anaemia were excluded. There was also a very small number of paired results with prenatal anaemia to be able to deduce that severe anaemia was indeed due to PPH. Without paired results postnatal severe anaemia was considered to be likely because of pre-existing anaemia and not sufficiently specific to PPH.

2. Heterogeneity is to be expected in results of test accuracy studies, thus random-effects models were used to describe the variability in test accuracy across studies. In the protocol, we had planned to examine subgroup and sensitivity results only by comparing the differences in prognostic sensitivities and specificities and prediction graphs. While some differences in sensitivities and specificities were observed they were subtle and difficult to interpret. As visual inspection of the prediction graphs across several subgroup characteristics only found methods of objective blood loss assessment as a key source of heterogeneity, we decided to compare the DOR between subgroups in determining important differences in prognostic performance of the clinical markers when used alone or in combination with other markers.

3. Sensitivity analysis based on removal of women without uterotonic prophylaxis could not be performed because only a third of women in the analysis had data on PPH prophylaxis.

3. Eligibility Criteria Checklist

Thank you for your interest in collaborating with the *Reappraisal of the Definition of Postpartum Haemorrhage* project. This form aims to map the availability of data on postpartum haemorrhage collected from the study of which you are the PI. Your response to this form is crucial to decide the suitability of your study and how we may pursue the data acquisition process.

***** Please answer one form per study if you have more than one study that you are willing to share *****

Completing this form will take about 10 minutes. Please send any queries you may have to PPHDefinitionProject@who.int with following email subject " Questions – Global call for data on postpartum haemorrhage".

We appreciate your interest in being part of this global initiative!

Personal information
For contact purposes

1.Full name

2.Email

Study information

3. Name of the study

4. Reference (DOI, if available)
Please consider the study protocol if the study has more than one reference

5.Were the data collected after a research ethics committee/board approval?
Yes [if yes, open Q6-9]
No

6. Please inform the number of the study approval in the research ethics committee/board.

7. Are you the data holder/provider for this study?
Yes
No

8. Please provide the contact information for the data holder for this study

9. Are you willing to share your data with the WHO under the terms described in the open call for data and according to the data-sharing agreement?
Please note that the dataset will be kept in a WHO repository located in Europe, with access restricted to the Steering group members and evidence synthesis team.
Yes [if yes, open Q10]
No

10. Does your institution have its own data sharing agreement?

Yes
No
Don't know

General details of the study

11.Please state the year of the data collection

12. Please state the country/countries where the data were collected

13. Please state the city where the data were collected

14. What were the eligibility criteria considered in the study?

15. What is the sample size of the study at enrolment?

16. Please state the study design

Observational

Experimental

17. *If this is a randomized control trial, what interventions were conducted among control/placebo groups?*

18. Does the dataset derives from public health registries or any other administrative registries from any local, national or international institution?

Yes

No

19. *If your answer was "Yes", please provide details of the data origin.*

20. Does the dataset report one or more of the following variables in women after childbirth: Postpartum blood loss, pulse rate, respiration rate, blood pressure, abnormal uterine tone, or any clinical signs and symptoms of haemodynamic instability?

Yes

No

21. Does the dataset report one or more of the following variables in women giving birth: Postpartum blood loss, pulse rate, respiration rate, blood pressure, abnormal uterine tone, or any clinical signs and symptoms of haemodynamic instability AND one or more of the following adverse clinical outcomes: Blood transfusion, administration of blood products (e.g. FFP, cryoprecipitate, fibrinogen concentrate, platelets) uterine tamponade, laparotomy, hysterectomy, uterine artery ligation, interventional radiology, cervical repair, postnatal anaemia, haemoglobin level or change in haemoglobin level post-birth or preoperative versus postoperative, maternal admission in high dependency unit, maternal ICU admission and maternal death?

Yes

No

22. Does the dataset report one or more of the following variables in women giving birth: Postpartum blood loss, pulse rate, respiration rate, blood pressure, abnormal uterine tone, or any clinical signs and symptoms of haemodynamic instability AND one or more of the following adverse clinical outcomes: Blood transfusion, administration of blood products (e.g. FFP, cryoprecipitate, fibrinogen concentrate, platelets), uterine tamponade, laparotomy, hysterectomy, uterine artery ligation, interventional radiology, cervical repair, postnatal anaemia, haemoglobin level or change in haemoglobin level post-birth or preoperative versus postoperative, maternal admission in high dependency unit, maternal ICU admission and maternal death?

Yes

No

4. Data Sharing Standards (DSS)

Data Structure

File format

- The preferred file formats are CSV, Excel, SPSS or Stata.
- Preferred unit of analysis: each record in the dataset must represent a unique visit per woman (i.e., the file should be in long format). If the dataset is in a wide format (i.e. one record contains information about multiple births per woman, please provide details on how this dataset could be converted into a long format).

Data Dictionary and Anonymization

Data dictionary

Please share a data dictionary to facilitate the harmonization of the dataset. This should be uploaded along with the dataset. This data dictionary should contain all variable names, descriptions, and value labels. For intervention trials, please provide information on the group allocations for participants.

Data anonymization

All datasets should be de-identified/anonymized before sharing with the WHO Steering Group on Postpartum Haemorrhage (PPH) Definition. **That is, no personally identifiable information, such as name, date of delivery, and geo-coordinates, should be included in the datasets shared.**

Overview of planned analyses and data sets

The initial planned analyses using these collated datasets are detailed below. These analyses are structured based on frameworks for developing evidence-based diagnostic parameters or thresholds for disease conditions. Other analyses not described here may be conducted at a future time within the remit of the project objectives. All analyses will be registered a priori using an online registration repository.

Analysis #1. Individual Participant Data (IPD) meta-analysis to produce distribution curves for postpartum blood loss and any other index tests or diagnostic parameters for PPH.

Aim: To synthesize and present the evidence on the distribution of blood loss and other index tests or diagnostic parameters (e.g. pulse, shock index etc.) after childbirth.

Type of datasets to be included: Studies that have reported one or more of the following variables in women after childbirth: Postpartum blood loss, pulse rate, respiration rate, blood pressure, abnormal uterine tone, or any clinical signs and symptoms of haemodynamic instability.

Analysis #2. Individual Participant Data (IPD) meta-analysis of datasets to produce distribution curves for postpartum blood loss and any other index tests or diagnostic parameters for PPH, separately for those with and without adverse clinical outcomes.

Aim: To synthesize and present the evidence on prognostic implications of various index tests for diagnosing PPH (e.g. amount of blood loss level, shock index, etc.).

Type of dataset to be included: Studies that have reported one or more of the following variables in women giving birth: Postpartum blood loss, pulse rate, respiration rate, blood pressure, abnormal uterine tone, or any clinical signs and symptoms of haemodynamic instability **AND** one or more of the following adverse clinical outcomes: Blood transfusion, administration of blood products (e.g. FFP, cryoprecipitate, fibrinogen concentrate, platelets) uterine tamponade, laparotomy, hysterectomy, uterine artery ligation, interventional radiology, cervical repair, postnatal anaemia,

haemoglobin level or change in haemoglobin level post-birth or preoperative versus postoperative, maternal admission in high dependency unit, maternal ICU admission and maternal death.

Analysis #3. Individual Participant Data (IPD) meta-analysis of datasets to evaluate the prognostic implications of (a) individual diagnostic test (type 2 prognostic factor research), (b) multiple factors (type 3 prognostic model research), and (c) therapeutic effects of diagnostic criteria and thresholds for PPH.

Aim: To synthesize and present the evidence on the prognostic implications of individual and combined tests or diagnostic parameters for PPH (e.g. blood loss, pulse, shock index etc.) after childbirth, as well as diagnostic criteria and thresholds for PPH on clinical outcomes after childbirth.

Type of dataset to be included: Studies that have reported one or more of the following variables in women giving birth: Postpartum blood loss, pulse rate, respiration rate, blood pressure, abnormal uterine tone, or any clinical signs and symptoms of haemodynamic instability **AND** one or more of the following adverse clinical outcomes: Blood transfusion, administration of blood products (e.g. FFP, cryoprecipitate, fibrinogen concentrate, platelets), uterine tamponade, laparotomy, hysterectomy, uterine artery ligation, interventional radiology, cervical repair, postnatal anaemia, haemoglobin level or change in haemoglobin level post-birth or preoperative versus postoperative, maternal admission in high dependency unit, maternal ICU admission and maternal death.

Predictor variables

The table below describes **desirable** predictor variables that could be included in the meta-analyses. Please provide all available data fields if they are present in your dataset. If you have any other variables not listed in the table that are relevant, we will also welcome these being submitted.

Name of the variable		Description	Type
**Maternal age**		Number of completed years, measured in the study or obtained from medical records	Continuous, nearest whole number (e.g. 32 years of age)
**Race or ethnic group**		Race category for participants; please provide the description of each group in the dataset	Categorical, one option only
**Pre-natal anaemia**	Ideal: Pre-natal Hb level	Hb in g/dL, measured in the study or obtained from medical records	Continuous, one decimal place (e.g. 9.8 g/dL)
	If Hb level is not available Acceptable: Diagnosis of pre-natal anaemia	Yes or No	Discrete, one option only
**Number of previous births**	All births	Number of births	Whole number
	Births ≥ 24 weeks		
**Blood group O**		Yes or No	Discrete, one option only

<table>
<tr><td colspan="2">History of inherited bleeding disorders</td><td>Yes or No</td><td>Discrete, one option only</td></tr>
<tr><td colspan="2">Previous history of PPH</td><td>Yes or No</td><td>Discrete, one option only</td></tr>
<tr><td colspan="2">Previous history of stillbirth</td><td>Yes or No</td><td>Discrete, one option only</td></tr>
<tr><td colspan="2">Previous history of caesarean section</td><td>Yes or No</td><td>Discrete, one option only</td></tr>
<tr><td colspan="2">Vaginal bleeding (antepartum haemorrhage) after 24 weeks of gestation or intrapartum haemorrhage in the index pregnancy</td><td>Yes or No</td><td>Discrete, one option only</td></tr>
<tr><td colspan="2">Diagnosis of pre-eclampsia in this pregnancy</td><td>Yes or No</td><td>Discrete, one option only</td></tr>
<tr><td colspan="2">Gestational age at birth</td><td>Number of completed weeks, measured in the study or obtained from medical records</td><td>Continuous, nearest whole number (e.g. 37 weeks)</td></tr>
<tr><td rowspan="3">Maternal Body Mass Index</td><td>Ideal: Maternal height</td><td>In centimetres, measured in the study or obtained from medical records</td><td>Continuous, nearest whole number (e.g. 162cm)</td></tr>
<tr><td>Ideal: Maternal weight</td><td>In kilograms, measured in the study or obtained from medical records</td><td>Continuous, nearest whole number (e.g. 56kg)</td></tr>
<tr><td>If maternal height and weight are not available

Acceptable: Body Mass Index</td><td>Body Mass Index calculated by:

$\frac{\text{Weight (kg)}}{[\text{Height (m)}]^2}$</td><td>Continuous, to one decimal place (e.g. 26.1)</td></tr>
<tr><td colspan="2">Did the woman have an induction or augmentation of labour?</td><td>Yes or No</td><td>Discrete, one option only</td></tr>
<tr><td colspan="2">Duration of labour</td><td>In minutes, measured in the study or obtained from medical records</td><td>Continuous, nearest whole number (e.g. 120 mins)</td></tr>
<tr><td colspan="2">Did the woman have an episiotomy?</td><td>Yes or No</td><td>Discrete, one option only</td></tr>
<tr><td colspan="2">Did the woman have a perineal tear?</td><td>Yes or No</td><td>Discrete, one option only</td></tr>
<tr><td colspan="2">Did the woman have a retained placenta for over 30 minutes or manual removal of placenta?</td><td>Yes or No</td><td>Discrete, one option only</td></tr>
<tr><td colspan="2">Birthweight</td><td>In grams, measured in the study or obtained from medical records</td><td>Continuous, nearest whole number (e.g. 3250g)</td></tr>
<tr><td colspan="2">Number of babies</td><td>Singleton, twin or triplet or higher order</td><td>Discrete, one option only</td></tr>
<tr><td colspan="2">Mode of birth</td><td>Spontaneous vaginal, forceps, ventouse, caesarean section</td><td>Discrete, one option only</td></tr>
<tr><td colspan="2" rowspan="3">Uterotonic used for prevention of PPH?</td><td>Oxytocin</td><td>Yes or No, one option only</td></tr>
<tr><td>Misoprostol</td><td>Yes or No, one option only</td></tr>
<tr><td>Ergometrine</td><td>Yes or No, one option only</td></tr>
</table>

	Carbetocin	Yes or No, one option only
	None given	Yes or No, one option only
**Any other relevant clinical variable**	If there are any other clinical variables not listed above, please provide these with the relevant data dictionary	

Test variables for diagnosing PPH

It is essential that at least one of the following test variables are included in the dataset. Please provide all available data fields if they are present in your dataset. If you have any other test variables not listed in the table that are relevant, we would also welcome these being submitted.

Name of the variable	Description	Type	Observations
**Abnormal uterine tone**	Yes or No, assessed during monitoring of the woman following birth	Discrete, one option only	
**Blood pressure**	Systolic pressure / diastolic pressure (mmHg), taken during monitoring of the woman following birth	Continuous, string variable (e.g. 120/80 mmHg)	
**Pulse rate**	Beats per minute, taken during monitoring of the woman following birth	Continuous, whole number (e.g. 75 bpm)	
**Respiration rate**	Breaths per specified time period (e.g. breaths per minute), taken during monitoring of the woman following delivery	Continuous, whole number (e.g. 16 breaths per minute)	
**Shock index score**	Shock index calculated by: $\frac{\text{Heart rate (bpm)}}{\text{Systolic blood pressure (mmHg)}}$	Continuous, to 1 decimal place (e.g. 1.3)	
**Chest pain**	Yes or no	Discrete, one option only	
**Altered mental state**	Yes or no	Discrete, one option only	
**Oliguria**	Yes or no	Discrete, one option only	
**Cool skin (due to reduced peripheral perfusion)**	Yes or no	Discrete, one option only	
**Peripheral oedema**	Yes or no	Discrete, one option only	
**Metabolic acidosis**	Yes or no	Discrete, one option only	
**Loss of consciousness**	Yes or no	Discrete, one option only	
**Postpartum blood loss**	In mL	Continuous, up to 2 decimal places (e.g. 330 ml)	

	Measurement method	Categorical, one option only	Weighed blood loss, measured with a calibrated drape, visual estimation.
**Any other relevant clinical variable**	If there are any other clinical variables not listed above, please provide these with the relevant data dictionary (e.g. any other clinical signs and symptoms of haemodynamic instability).		

Dependent variables

Name of the variable		Description	Type
**Additional uterotonics**		Yes or No	Discrete, one option only
**Whole blood transfusion**		Yes or No	Discrete, one option only
**Blood product transfusion**		Yes or No	Discrete, one option only
**Uterine tamponade**		Yes or No	Discrete, one option only
**Laparotomy**		Yes or No	Discrete, one option only
**Uterine artery ligation**		Yes or No	Discrete, one option only
**Internal iliac artery ligation**		Yes or No	Discrete, one option only
**B-Lynch suture**		Yes or No	Discrete, one option only
**Interventional radiology**		Yes or No	Discrete, one option only
**Hysterectomy**		Yes or No	Discrete, one option only
**Cervical repair**		Yes or No	Discrete, one option only
**Postnatal anaemia**	Ideal: Hb level post-birth	Hb in g/dL, measured in the study or obtained from medical records	Continuous, one decimal place (e.g. 9.8 g/dL)
	If Hb level is not available<br><br>Acceptable: Diagnosis of postnatal anaemia	Yes or No	Discrete, one option only, please provide the Hb level to diagnose anaemia (g/dL)
**Maternal sepsis**		Yes or No	Discrete, one option only
**Maternal admission in high dependency unit**		Yes or No	Discrete, one option only
**Maternal ICU admission**		Yes or No	Discrete, one option only
**Vital organ failure (temporary or permanent)**		Yes or No	Discrete, one option only
**Maternal death**		Yes or No	Discrete, one option only
**Any other relevant clinical variable**		If there are any other clinical variables not listed above, please provide these with the relevant data dictionary	

Data Sharing and Security Procedures

Information security and privacy policy

Information security at WHO is based on the ISO 27001 standard. WHO has formal and comprehensive information security policies with respective standard operating procedures. Policies cover information security, access to information and systems, cloud computing, application security,

information classification, and related security standards. For more details on WHO's five core data principles, please visit https://www.who.int/data/principles. WHO's data policy can be found here https://www.who.int/about/policies/publishing/data-policy.

In its mission to uphold member states' trust in data, WHO ensures that all data shared with the organization are securely and confidentially stored. WHO also upholds the highest data protection standards and respect for human rights, including the right to privacy, about any personal data and data aggregates of groups of individuals in WHO-controlled data sets. Before sharing any datasets with the organization, we require member States and non-State actors who share data to confirm that the data have been collected following applicable national laws, including data protection laws to protect the confidentiality of identifiable persons.

Repository details

All the microdata sets that will be shared will be housed in a WHO-managed SharePoint environment. SharePoint is an online, secure document repository developed and maintained by Microsoft. This document repository is private and compliant with General Data Protection Regulation (GDPR).

There are three levels in the Postpartum Haemorrhage (PPH) Repository. The first two levels (orange and green) are only accessible to the WHO steering group and WHO statisticians. WHO staff not working on the PPH project and other researchers that supply data to the project will not get access to these levels.

The third level (blue, purple, and red) is only accessible to the study's principal investigator (PI) or data provider supplying data to the PPH definition project. Data providers or PI will only have access to folders related to the study shared. They will not have access to the folder in other studies. For example, the data provider given access to the blue folder will not get access to the purple or red folders even though they are on the same level.

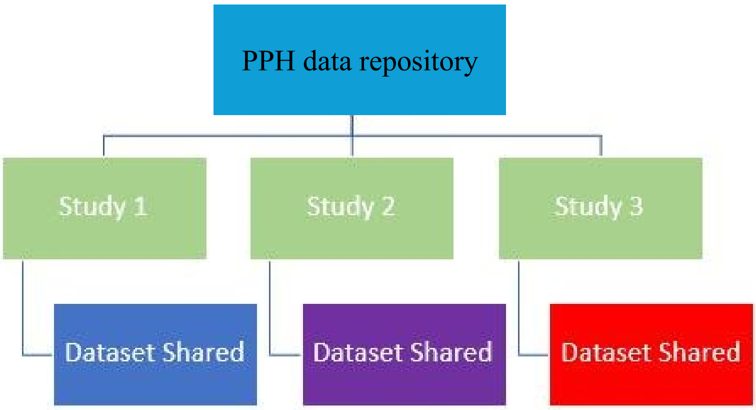

Data upload

Stage 1: *Signing data sharing agreement*

After a study (dataset) has met the eligibility criteria, the WHO and data provider will complete a data sharing agreement that permits the WHO steering group to use the microdata for the purpose of reappraising the PPH definition. The data agreement will contain the corresponding data providers' names, affiliations, and e-mails and a confirmation that the data has been collected following applicable national laws, including data protection laws and ethical standards.

Stage 2: *Shared folder invitation*

Upon submission of the data agreement, the corresponding data provider(s) will receive an e-mail invitation from the WHO steering group inviting the data provider to their specific data folder. The data provider will have to click "Open" to proceed to the next stage.

Stage 3: *Requesting a verification link*

Clicking "Open" in Stage 2 will navigate the data provider to a webpage where they can request a verification code to access the Shared Folder.

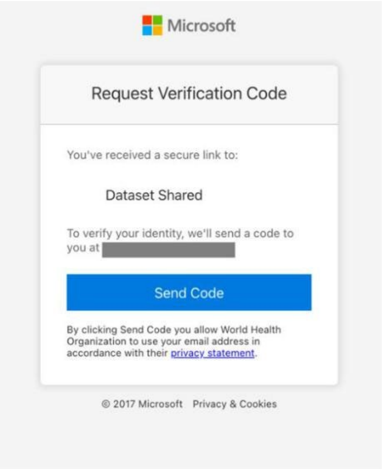

After clicking "Send Code", the data provider will receive an e-mail from no-reply@notify.microsoft.com with a verification code. That e-mail will resemble the image below:

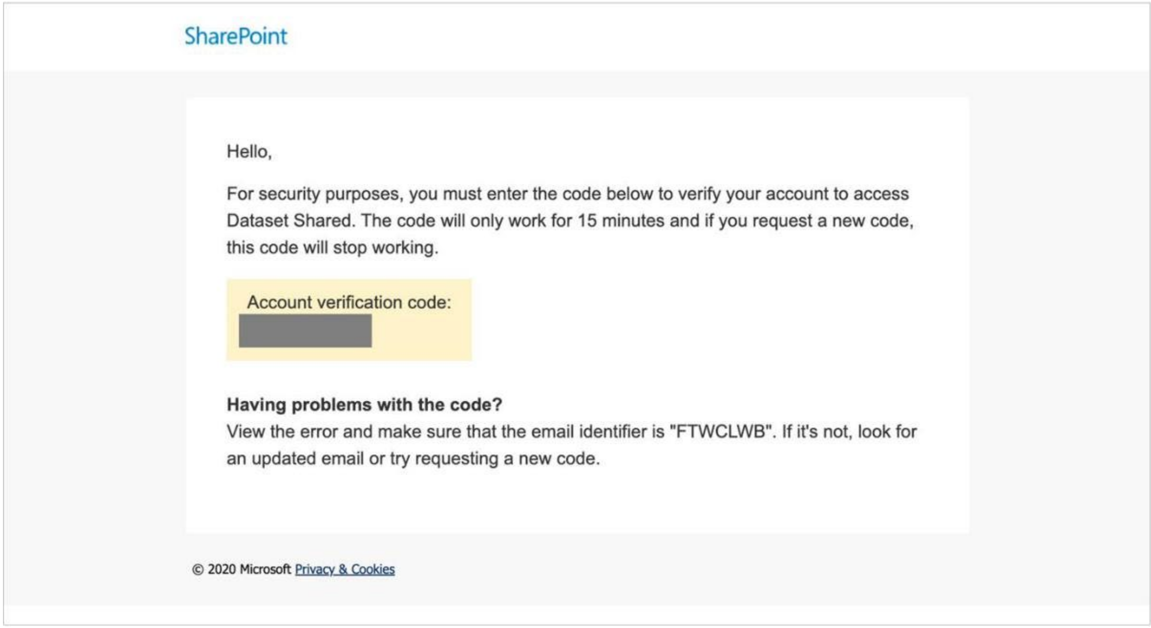

The verification codes are time-bound and will only work for 15 minutes. The data provider will have to request a new code every time you sign into the folder.

Stage 4: *Uploading a dataset*

Once the data provider has access to the folder, they can upload the dataset by selecting "Upload" and selecting the file from the location from their local device.

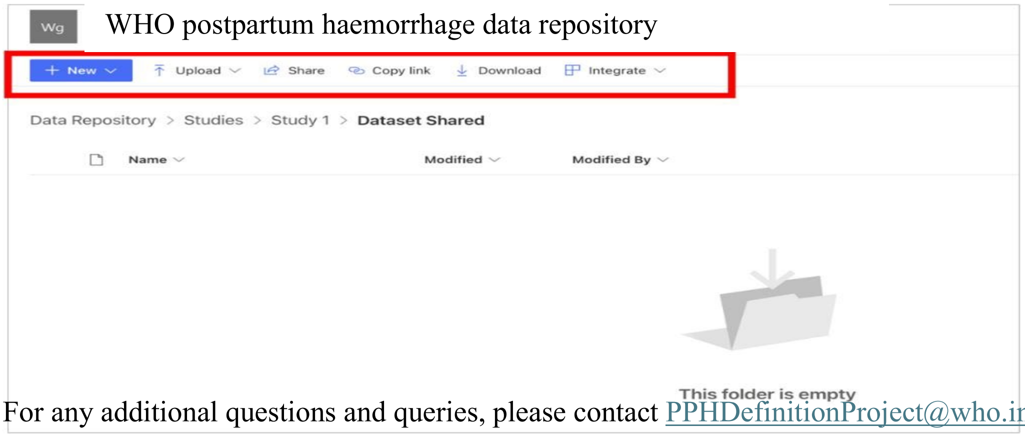

For any additional questions and queries, please contact PPHDefinitionProject@who.int.

5. Supplementary Results

Table S1: Included Studies in IPD Meta-Analysis

Study	Title	Total N	Objectives	Design	Setting	Participants	Tests	Outcomes
**Devall 2025**[1]	Implementation study of Early Detection and Treatment of Postpartum Haemorrhage	17 890	To determine if early diagnosis and treatment of PPH can be implemented in South Asia setting	Before and after implementation study	Four secondary-level hospitals in Pakistan.	Women giving birth vaginally; hospitals practiced early detection and bundle of first response treatment or usual care.	Blood loss was measured using a drape for up to two hours and was weighed on a digital scale.	Blood transfusion, surgical procedures (hysterectomy, laparotomy), admission to intensive care unit, maternal death.
**Durocher 2019**[2]	Does route matter? Impact of route of oxytocin administration on postpartum bleeding: A double-blind, randomized controlled trial	480	To determine the effectiveness of IV versus IM prophylactic oxytocin	Randomized controlled trial	One hospital in Argentina.	Women giving birth vaginally were randomized to receive oxytocin IV or IM.	Blood loss was measured using a calibrated drape for one hour postpartum.<br><br>Blood pressure was reported at 15, 30, 45, and 60 minutes after birth.<br><br>Pulse was reported at 15, 30, 45, and 60 minutes after birth.	Blood transfusion, surgical procedures (hysterectomy, laparotomy), maternal death.
**Gallos 2023**[3]	Randomized Trial of Early Detection and Treatment of Postpartum Haemorrhage	210 132	To determine if early diagnosis and treatment of PPH is more effective than usual care	Cluster randomized controlled trial	80 secondary-level hospitals in Kenya, Nigeria, South Africa, and Tanzania.	Women giving birth vaginally; hospitals were randomized to early detection and bundle of first response treatment or usual care.	Blood loss was measured using a drape for up to two hours and was weighed on a digital scale.	Blood transfusion, surgical procedures (hysterectomy, laparotomy), admission to intensive care unit, maternal death.
**Gülmezoglu 2001**[4]	Multicentre randomized trial of misoprostol in the management of the third stage of labour.	18 530	To determine if prophylactic oral misoprostol is as effective as oxytocin	Randomized controlled trial	Hospitals in Argentina, China, Egypt, Ireland, Nigeria, South Africa, Switzerland, Thailand, and Vietnam.	Women giving birth vaginally were randomized to prophylactic misoprostol or oxytocin	Blood loss was measured using a flat bedpan and a measuring jar for one hour postpartum.<br><br>Blood pressure was reported as the lowest value within one hour postpartum.	Blood transfusion, hysterectomy, admission to intensive care unit, and maternal death.
**Gülmezoglu 2012**[5]	Active management of the third stage of labour with and without controlled cord traction: a randomized, controlled, non-inferiority trial.	24 390	To determine if prophylactic oxytocin is as effective as the full package of active management of third stage	Randomized controlled trial	16 hospitals and two primary healthcare centres in Argentina, Egypt, India, Kenya, the Philippines,	Women giving birth vaginally were randomized to immediate or delayed controlled cord traction. All women received 10 IU of prophylactic oxytocin.	Blood loss was measured using a drape and was weighed on a digital scale.	Blood transfusion, surgical procedures (hysterectomy, ligation of vessels), admission to intensive care unit, maternal death.

					South Africa, Thailand, and Uganda.			
**Haslinger 2020**[6]	The impact of prepartum factor XIII activity on postpartum blood loss.	1 309	To determine whether coagulation factors, and in particular prepartum FXIII activity, influence postpartum blood loss in previously asymptomatic women	Prospective cohort	One hospital in Switzerland.	677 women who gave birth vaginally and 632 women who gave birth by caesarean section.	Blood loss was measured using a combination of gravimetric and volumetric approaches.	Blood transfusion.
**Mammoliti 2025**[7]	Nested observational cohort within the randomized Trial of Early Detection and Treatment of Postpartum Haemorrhage	5 412 *(nested within 210 132 from Gallos 2023)*	To explore fidelity of implementation for the early diagnosis and treatment of PPH	Nested observational cohort within a cluster trial	80 secondary-level hospitals in Kenya, Nigeria, South Africa, and Tanzania.	5 412 women giving birth vaginally were selected randomly within a cluster trial where hospitals were randomized to early detection and bundle of first response treatment or usual care.	Blood loss was measured using a drape for up to two hours and was weighed on a digital scale.<br><br>Blood pressure was reported at 15, 30, 45, and 60 minutes after birth.<br><br>Pulse was reported at 15, 30, 45, and 60 minutes after birth.	Blood transfusion, surgical procedures (hysterectomy, laparotomy), admission to intensive care unit, maternal death.
**Mobeen 2011**[8]	Administration of misoprostol by trained traditional birth attendants to prevent postpartum haemorrhage in homebirths in Pakistan: a randomized placebo-controlled trial	1119	To determine safety and effectiveness of prophylactic oral misoprostol by traditional birth attendants	Randomized controlled trial	Remote villages of Chitral, Khyber Pakhtunkhwa Province, Pakistan.	Women giving birth vaginally were randomized to receive prophylactic misoprostol or placebo.	Women were positioned on a perineal sheet and bedpan for a minimum of 1 hour. Collected blood was poured into a jar, sealed, and weighed 24-48 hrs postpartum by a trained health worker.	Referral to a higher level of care if blood transfusion or surgical procedures (hysterectomy) were required, maternal death.
**Pacagnella 2021**[9]	The golden hour for postpartum haemorrhage	270	To evaluate the predictive capacity of vital signs for the diagnosis of PPH	Prospective cohort	One hospital in Brazil.	270 women giving birth vaginally. All received standard prophylaxis with oxytocin.	Blood loss was measured using a drape and was weighed on a digital scale.<br><br>Blood pressure, pulse, respiratory rate, and shock index were measured every five minutes up to 30 minutes, every 15 minutes up to two hours postpartum, and then “routinely” up to 24 hours postpartum.	Blood transfusion, surgical procedures (hysterectomy), maternal death.

**Sentilhes 2018**[10]	Tranexamic acid for the prevention of blood loss after vaginal delivery	3891	To determine the effectiveness of prophylactic TXA at CS in addition to standard care	Randomized controlled trial	15 hospitals in France.	Women giving birth vaginally were randomized to receive prophylactic tranexamic acid or placebo in addition to standard prophylaxis with oxytocin.	Blood loss was measured using a calibrated drape for 15 minutes postpartum, or until the birth attendant considered bleeding to have stopped.<br><br>Blood pressure was measured at 15, 30, 45, and 60 minutes after birth and reported the lowest systolic pressure. Pulse was reported as the pulse occurring with the lowest systolic pressure.<br><br>Shock index was reported as the highest index after birth.	Blood transfusion, surgical procedures (hysterectomy, ligation of vessels), admission to intensive care unit, maternal death.
**Sentilhes 2021**[11]	Tranexamic acid for the prevention of blood loss after caesarean delivery	4431	To determine the effectiveness of prophylactic TXA at caesarean birth in addition to standard care	Randomized controlled trial	27 hospitals in France.	Women giving birth by caesarean section were randomized to receive prophylactic tranexamic acid or placebo, all received standard prophylaxis with oxytocin.	Blood loss was measured gravimetrically for two hours postpartum, or until the birth attendant considered bleeding to have stopped.<br><br>Blood pressure was measured at 15, 30, 45, and 60 minutes after birth and reported the lowest systolic pressure. Pulse was reported as the pulse with the lowest systolic pressure.<br><br>Shock index was reported as the highest index after birth.	Blood transfusion, surgical procedures (hysterectomy, ligation of vessels), admission to intensive care unit, maternal death.
**Widmer 2018**[12]	Heat-stable carbetocin versus oxytocin to prevent haemorrhage after vaginal birth.	29 645	To determine if prophylactic heat stable carbetocin is as effective as oxytocin	Randomized controlled trial	23 hospitals in Argentina, Egypt, India, Kenya, Nigeria, Singapore, South Africa, Thailand, Uganda, and the United Kingdom.	Women giving birth vaginally were randomized to prophylactic carbetocin or oxytocin	Blood loss was measured using a drape and was weighed on a digital scale.	Blood transfusion, surgical procedures (hysterectomy, ligation of vessels), admission to intensive care

1. Devall A. Early detection and bundled treatment of postpartum haemorrhage with the E-MOTIVE intervention: A prospective pre-post intervention study in Pakistan. Unpublished data.; 2. Durocher J, Dzuba IG, Carroli G, Morales EM, Aguirre JD, Martin R, et al. Does route matter? Impact of route of oxytocin administration on postpartum bleeding: A double-blind, randomized controlled trial. PLoS ONE. 2019 Oct 1;14(10):e0222981.; 3. Gallos I, Devall A, Martin J, Middleton L, Beeson L, Galadanci H, et al. Randomized trial of early detection and treatment of postpartum hemorrhage. New England Journal of Medicine. 2023 May 9;389(1):11–21.;4. Gülmezoglu AM, Villar J, Ngoc NTN, Piaggio G, Carroli G, Adetoro L, et al. WHO multicentre randomised trial of misoprostol in the management of the third stage of labour. The Lancet. 2001 Sep 1;358(9283):689–95.;

5. Gülmezoglu AM, Lumbiganon P, Landoulsi S, Widmer M, Abdel-Aleem H, Festin M, et al. Active management of the third stage of labour with and without controlled cord traction. Obstetrical & Gynecological Survey. 2012 Sep;67(9):531–2.; 6. Haslinger C, Korte W, Hothorn T, Brun R, Greenberg C, Zimmermann R. The impact of prepartum factor XIII activity on postpartum blood loss. Journal of Thrombosis and Haemostasis. 2020 Jun;18(6):1310–9.; 7. Mammoliti KM. When are postpartum haemorrhages detected? A nested observational study within the E-MOTIVE cluster-randomised trial. Unpublished data.; 8. Mobeen N, Durocher J, Zuberi N, Jahan N, Blum J, Wasim S, et al. Administration of misoprostol by trained traditional birth attendants to prevent postpartum haemorrhage in homebirths in Pakistan: A randomised placebo-controlled trial. BJOG: An International Journal of Obstetrics & Gynaecology. 2010 Dec 23;118(3):353–61.; 9. Pacagnella RC, Borovac-Pinheiro A, Silveira C, Siani Morais S, Argenton JLP, Souza JP, et al. The golden hour for postpartum hemorrhage: Results from a prospective cohort study. International Journal of Gynecology & Obstetrics. 2021 Jul 22;(0):1–9.; 10. Sentilhes L, Winer N, Azria E, Sénat MV, le Ray C, Vardon D, et al. Tranexamic acid for the prevention of blood loss after vaginal delivery. New England Journal of Medicine. 2018 Aug 23;379(8):731–42.; 11. Sentilhes L, Sénat M v., le Lous M, Winer N, Rozenberg P, Kayem G, et al. Tranexamic acid for the prevention of blood loss after cesarean delivery. New England Journal of Medicine. 2021 Apr 29;384(17):1623–34.; 12. Widmer M, Piaggio G, Nguyen TMH, Osoti A, Owa OO, Misra S, et al. Heat-stable carbetocin versus oxytocin to prevent hemorrhage after vaginal birth. New England Journal of Medicine. 2018 Jun 27;379(8):743–52.

Table S2: Summary of Clinical Characteristics of Participants

	Number (%) or median (IQR)	Participants with data available for variable
**Maternal characteristics**		
Maternal age (years)	26·0 (22·0–30·2)	308 206
Pre-natal haemoglobin (g/dL)	11·9 (11·0–12·7)	14 287
Number of previous births	1·0 (0·0–3·0)	280 527
Previous history of PPH	3 072 (1·2%)	247 854
Previous caesarean	9 306 (3·7%)	252 550
Gestational age	38·0 (37·0–40·0)	288 637
Induction or augmentation of labour	59 339 (22·1%)	268 007
Duration of labour (min.)	285·5 (179·0–458·0)	4 656
Episiotomy	35 121 (15·1%)	232 490
Perineal tear	66 611 (25·9%)	257 072
Retained placenta	5 438 (1·7%)	311 607
Birthweight	3 080 (2 800–3 400)	310 134
Number of babies		
Singleton	258 153 (98·4%)	262 455
Multiple	4 302 (1·6%)	262 455
Mode of birth		
Spontaneous vaginal	299 435 (96·0%)	312 047
Instrumental vaginal	6 674 (2·1%)	312 047
Caesarean	5 938 (1·9%)	312 047
Prophylactic uterotonic [a]		
Oxytocin or carbetocin	95 396 (90·0%)	106 041
Misoprostol	21 580 (20·4%)	106 041
None given	995 (0·9%)	106 041
**Clinical markers**		
Measured blood loss (mL)	200 (120–349)	305 523
Pulse rate (bpm)	85 (76–96)	12 765
Systolic blood pressure (mmHg)	110 (105–120)	31 616
Diastolic blood pressure (mmHg)	70 (60–80)	31 609
Shock Index score	0·81 (0·72–0·94)	12 860
**Adverse PPH-related outcomes**		
Additional uterotonics	35 058 (11·3%)	310 321
Composite morbidity and mortality outcome [b]	7 769 (2·5%)	311 994
Blood or blood products transfusion	7 223 (2·3%)	311 898
Morbidity [c]	253 (0·08%)	310 316
Surgery [d]	641 (0·21%)	310 621
Maternal death	126 (0·04)	309 064

[a] Some women received more than one prophylactic uterotonic.
[b] For participants that suffered multiple components of the composite were only counted once for the purpose of this analysis.
[c] Morbidity included maternal sepsis (2 cases), ICU admission (205 cases), hysterectomy (71 cases) and high-care transfers (18 cases). Data on high-dependency unit admission and organ failure were not consistently reported.
[d] Surgery included uterine tamponade (292 cases), laparotomy with uterine artery ligation (19 cases), laparotomy with internal iliac artery ligation (9 cases), laparotomy with B-Lynch suture (271 cases), and interventional radiology (12 cases), and hysterectomy (71 cases).

Maternal race/ethnicity and blood group O not reported; information on additional maternal characteristics such as maternal BMI, history of stillbirth, and history of bleeding disorders were sought but there were insufficient data for analysis; additional tests such as respiratory rate, chest pain, altered mental state, cool skin, and loss of consciousness were sought but there were insufficient data for analysis.

Figure S1. Risk of Bias Assessment.

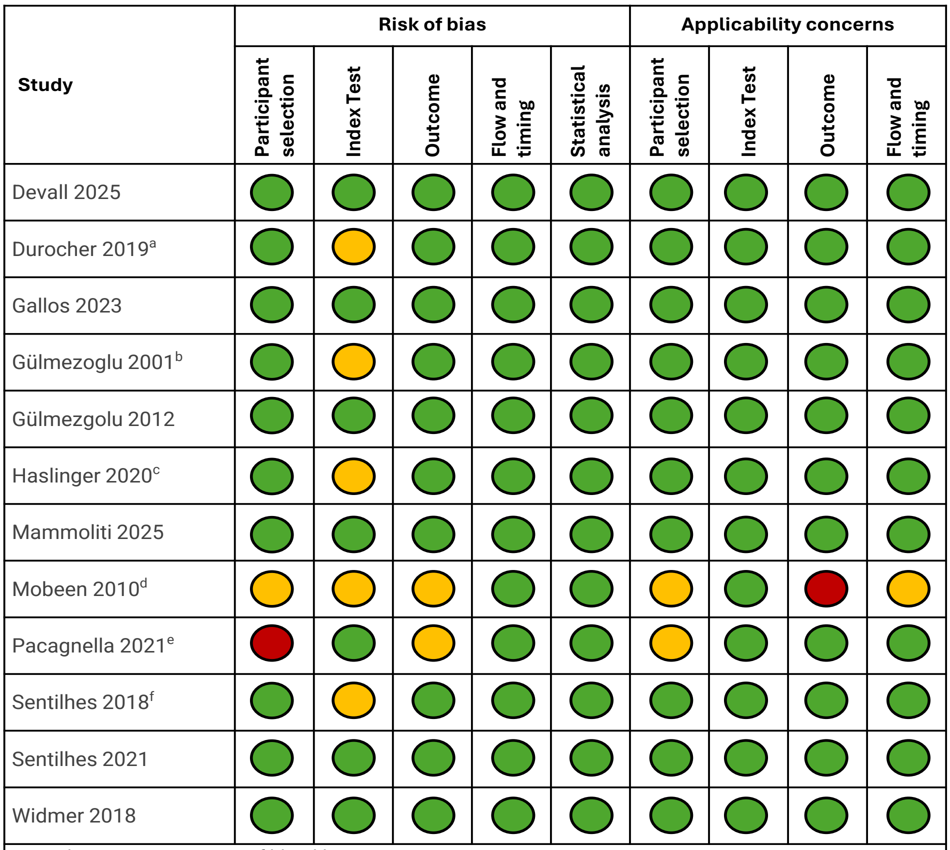

a – Volumetric assessment of blood loss.
b – Volumetric assessment of blood loss.
c – Volumetric assessment of blood loss.
d – Possible selection bias, volumetric assessment of blood loss, unclear measurement of outcome (referral); concerns about representativeness of sample, concerns about applicability of study outcome (referral) to IPD outcome (composite), lack of clarity around time horizons.
e – Convenience sample and possible selection bias, lack of clarity about measurement of outcomes; concerns about representativeness of sample.
f – Volumetric assessment of blood loss.

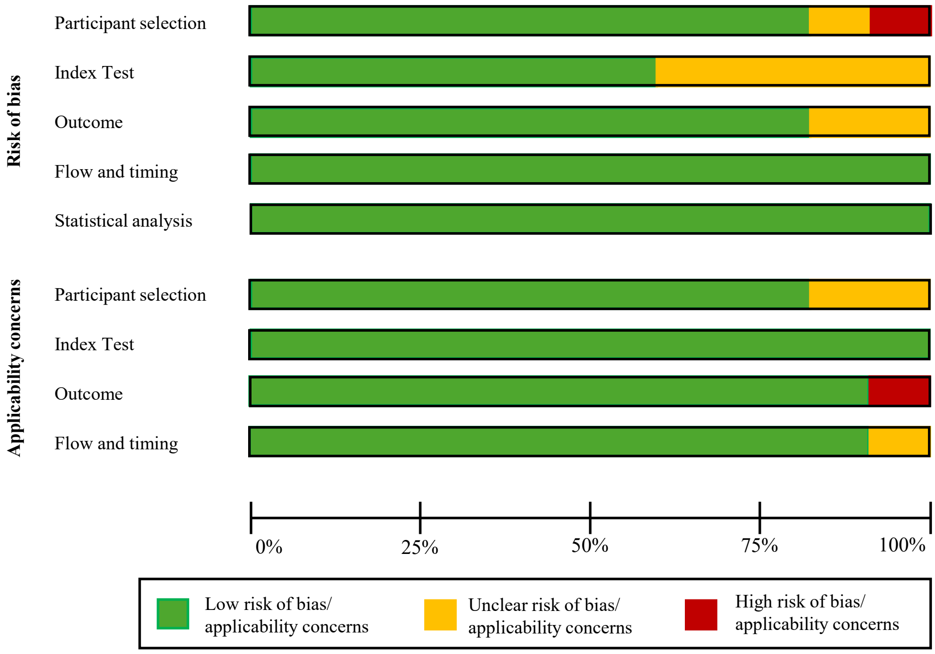

Figures S2-S6. Summary ROC Plots for Individual Markers

Figure S2. Summary ROC plots for blood loss thresholds in predicting death or severe morbidity.

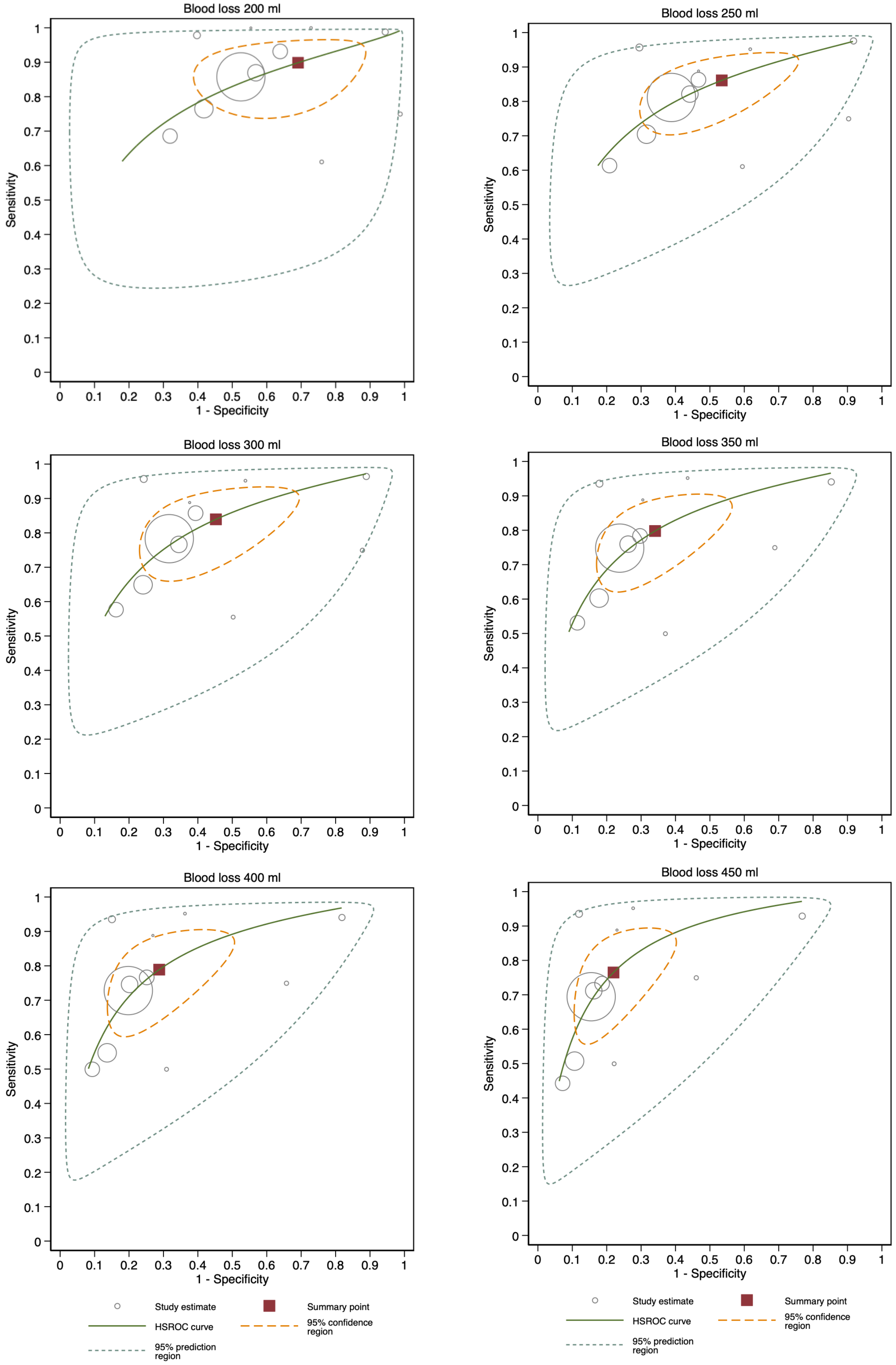

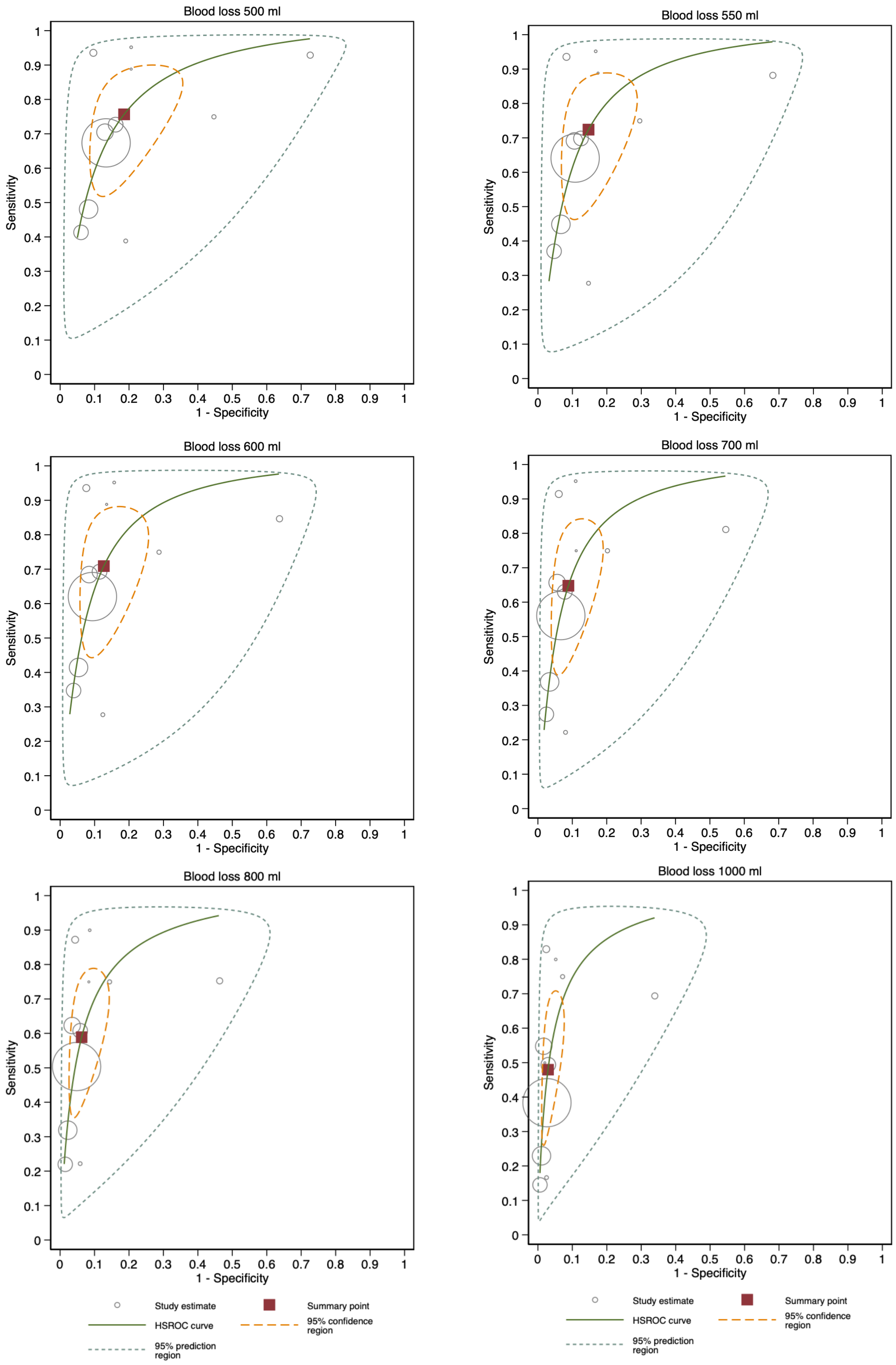

* Figures are based on analysis of 305 523 women.

Figure S3. Summary ROC plots for systolic blood pressure thresholds in predicting death or severe morbidity.

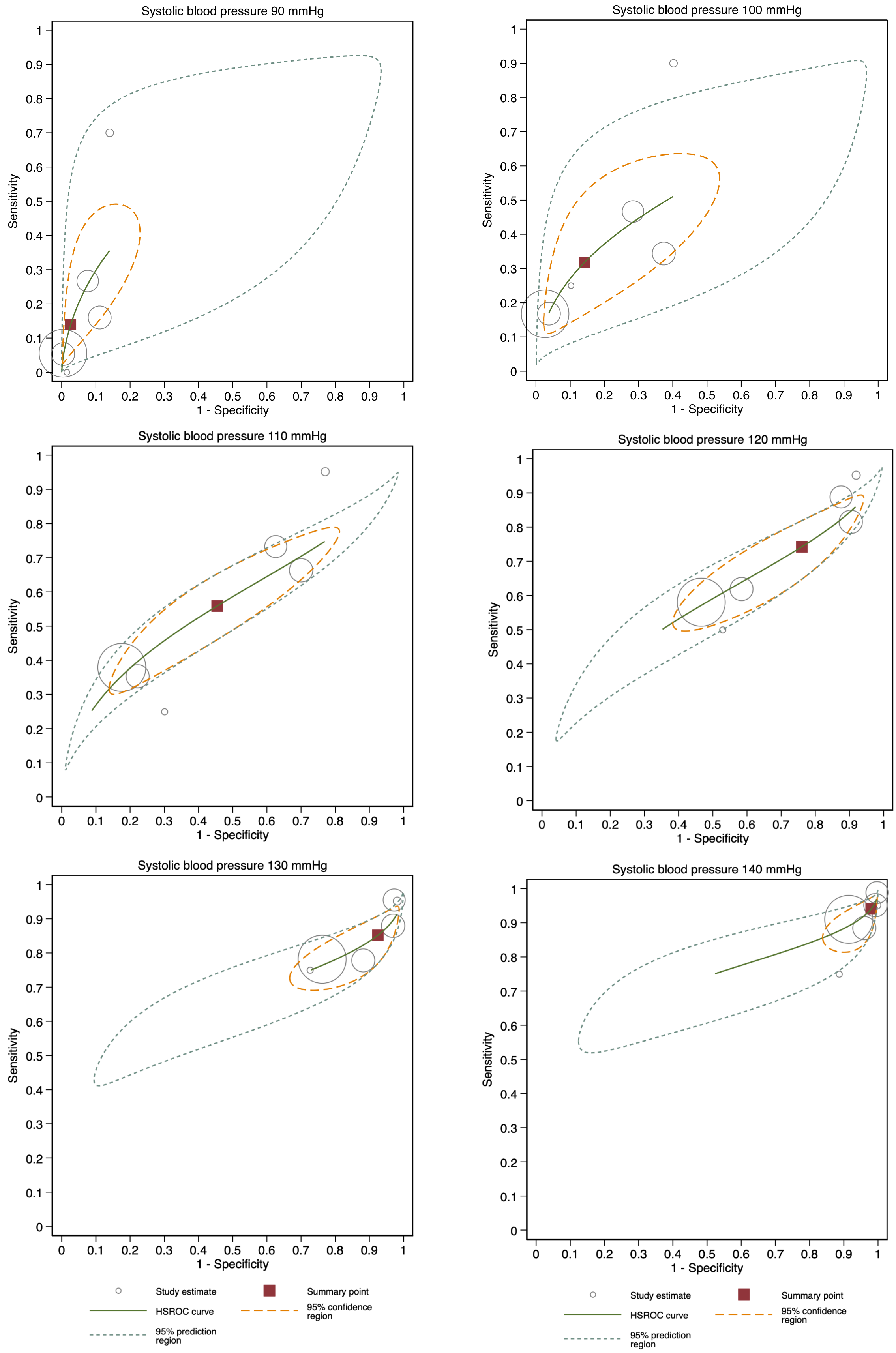

[*] Figures are based on analysis of 31 567 women.

Figure S4. Summary ROC curve for diastolic blood pressure thresholds in predicting death or severe morbidity.

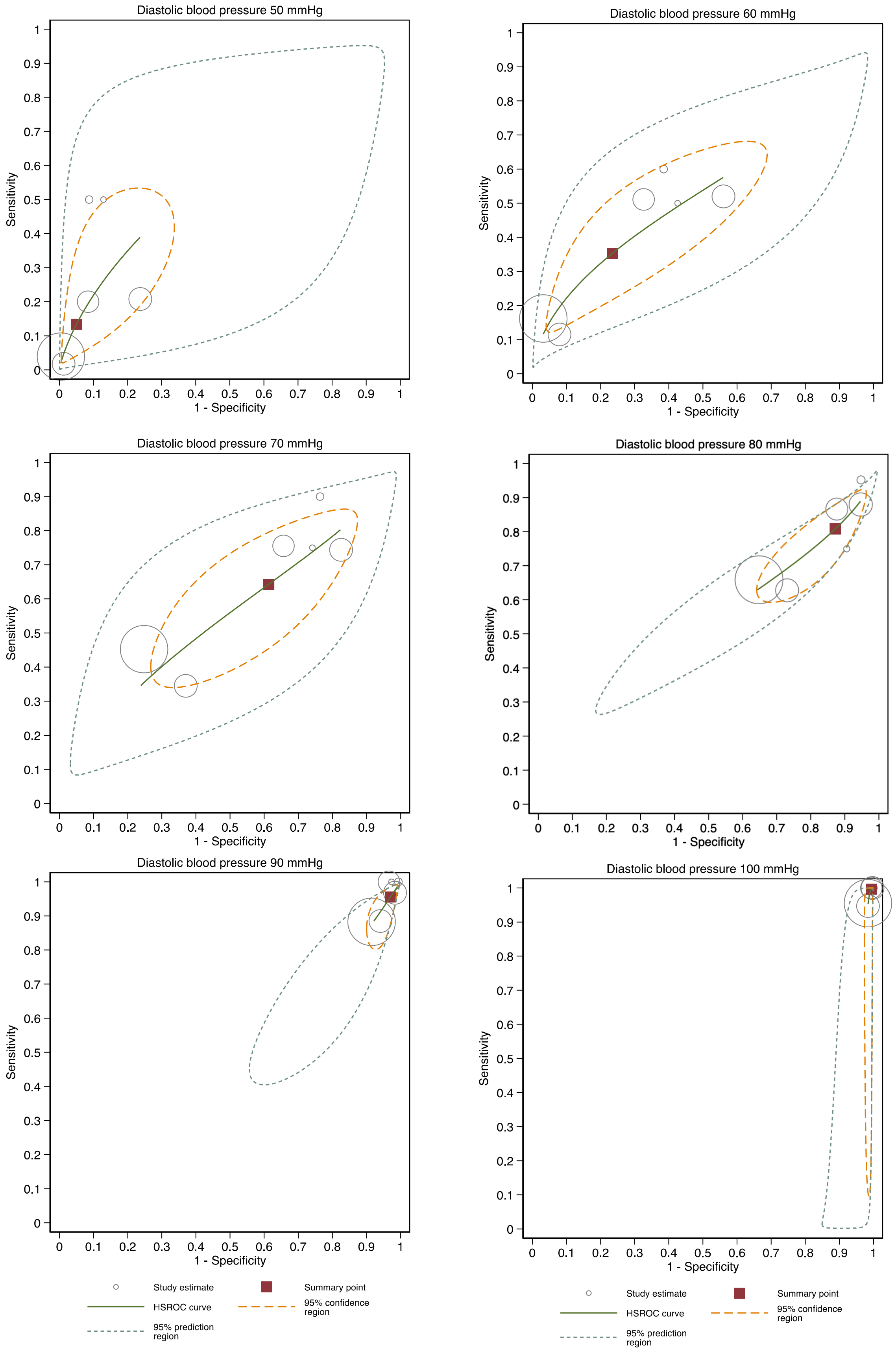

* Figures are based on analysis of 31 560 women.

Figure S5. Summary ROC curve for pulse thresholds in predicting death or severe morbidity.

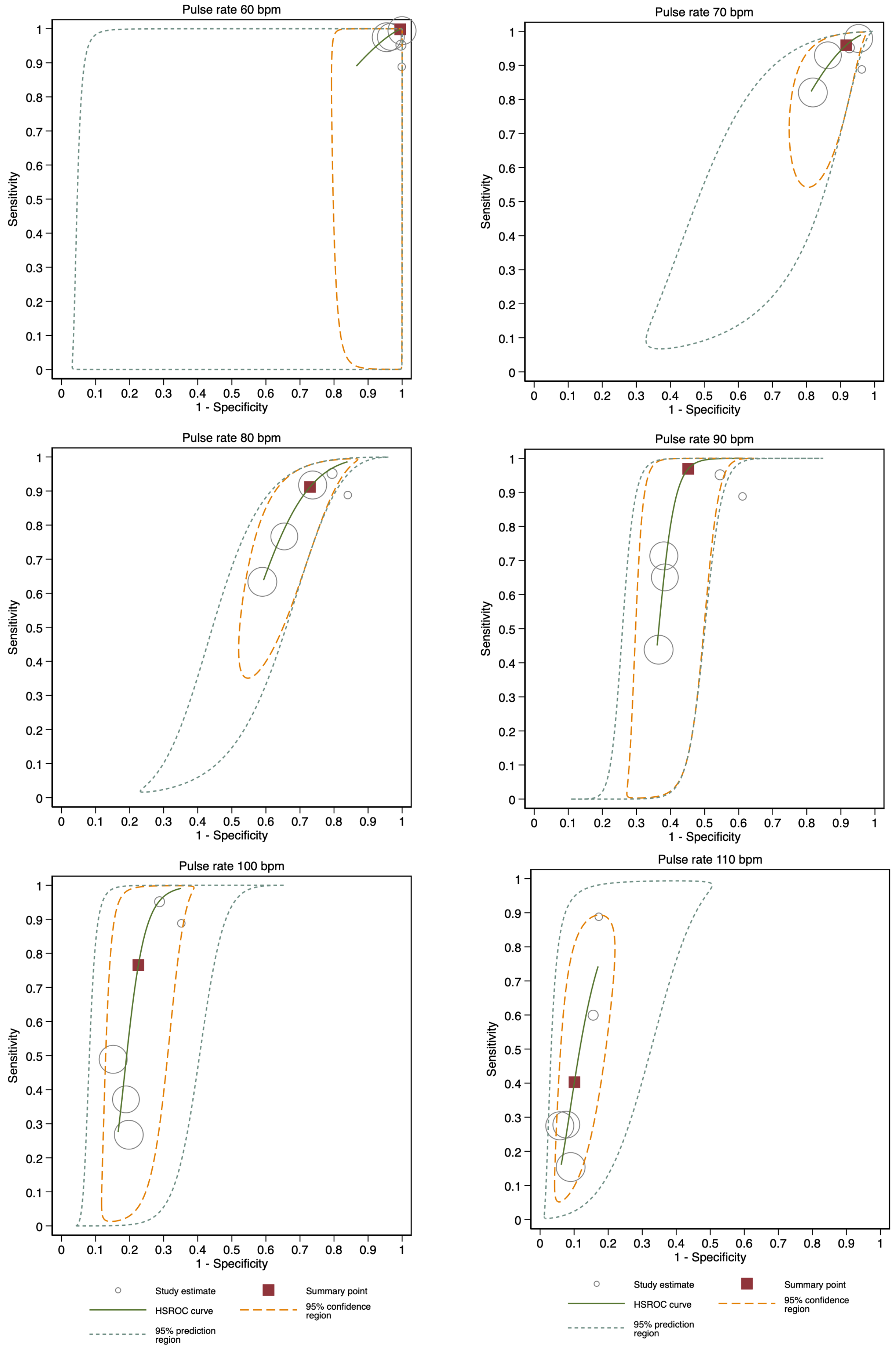

[*] Figures are based on analysis of 12 716 women.

Figure S6. Summary ROC curve for shock index thresholds in predicting death or severe morbidity.

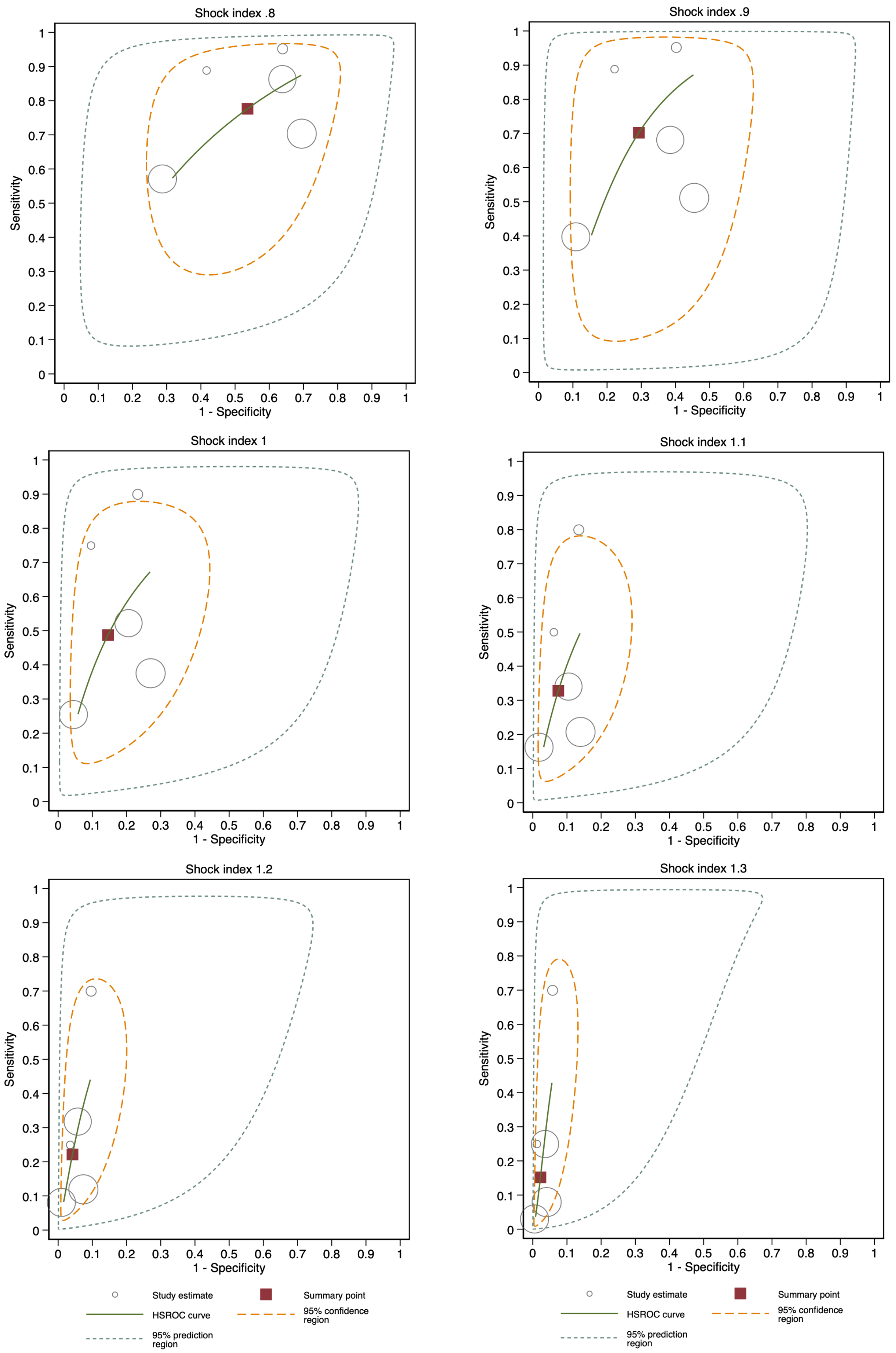

[*] Figures are based on analysis of 12 716 women.

Figures S7-S9. Summary ROC Plots for Decision Rules

Figure S7. Summary ROC curve for blood loss thresholds in combination with haemodynamic signs in predicting maternal death or severe morbidity (Decision rule 1: women considered at high risk when either blood loss exceeded threshold or any haemodynamic sign was abnormal).

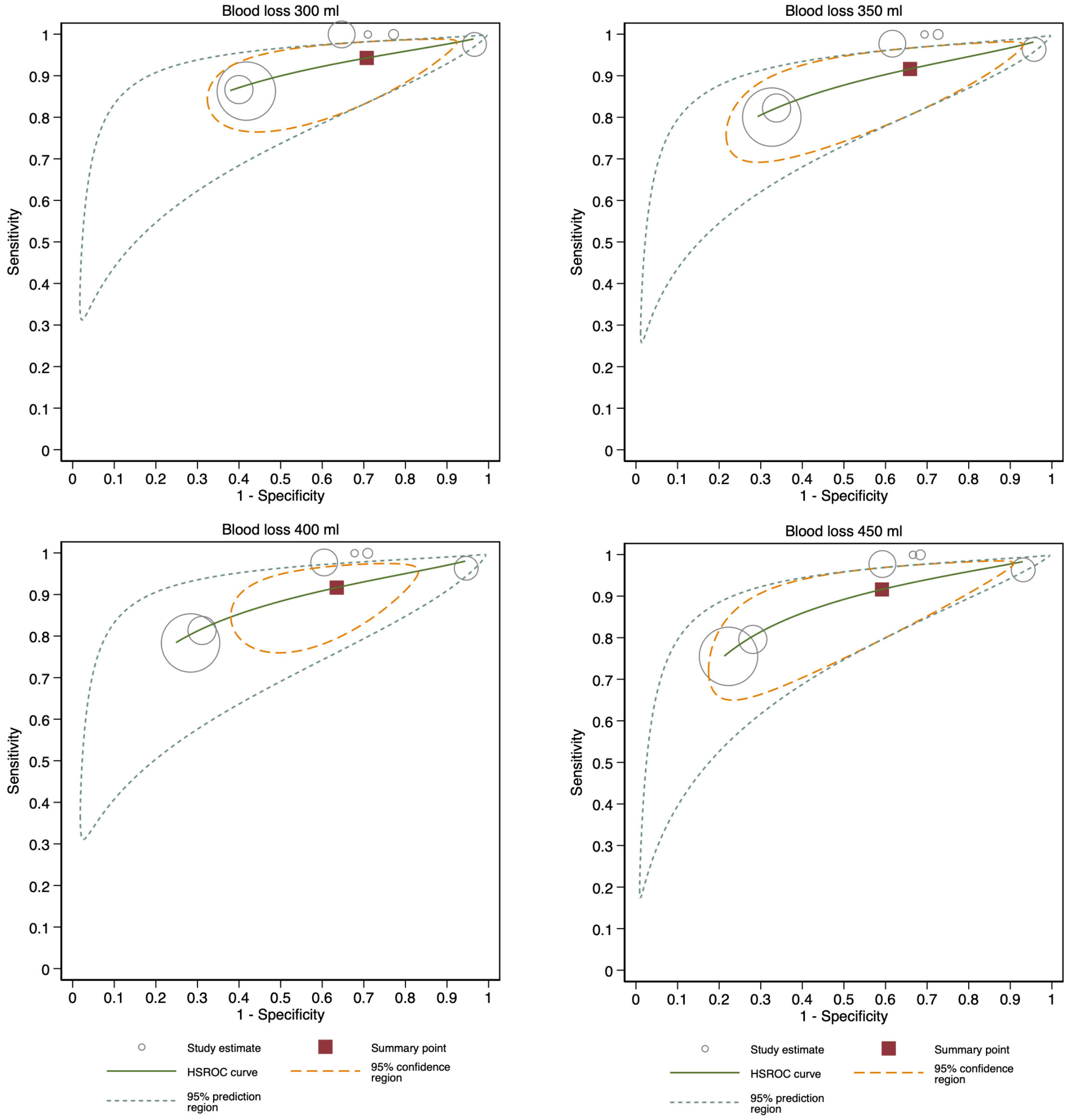

[*] Rules 1 to 3 are based on analysis of 30 691 women.

Figure S8. Summary ROC curve for blood loss thresholds in combination with haemodynamic signs in predicting maternal death or severe morbidity (Decision rule 2: women considered at high risk when both blood loss exceeded threshold and any haemodynamic sign was abnormal).

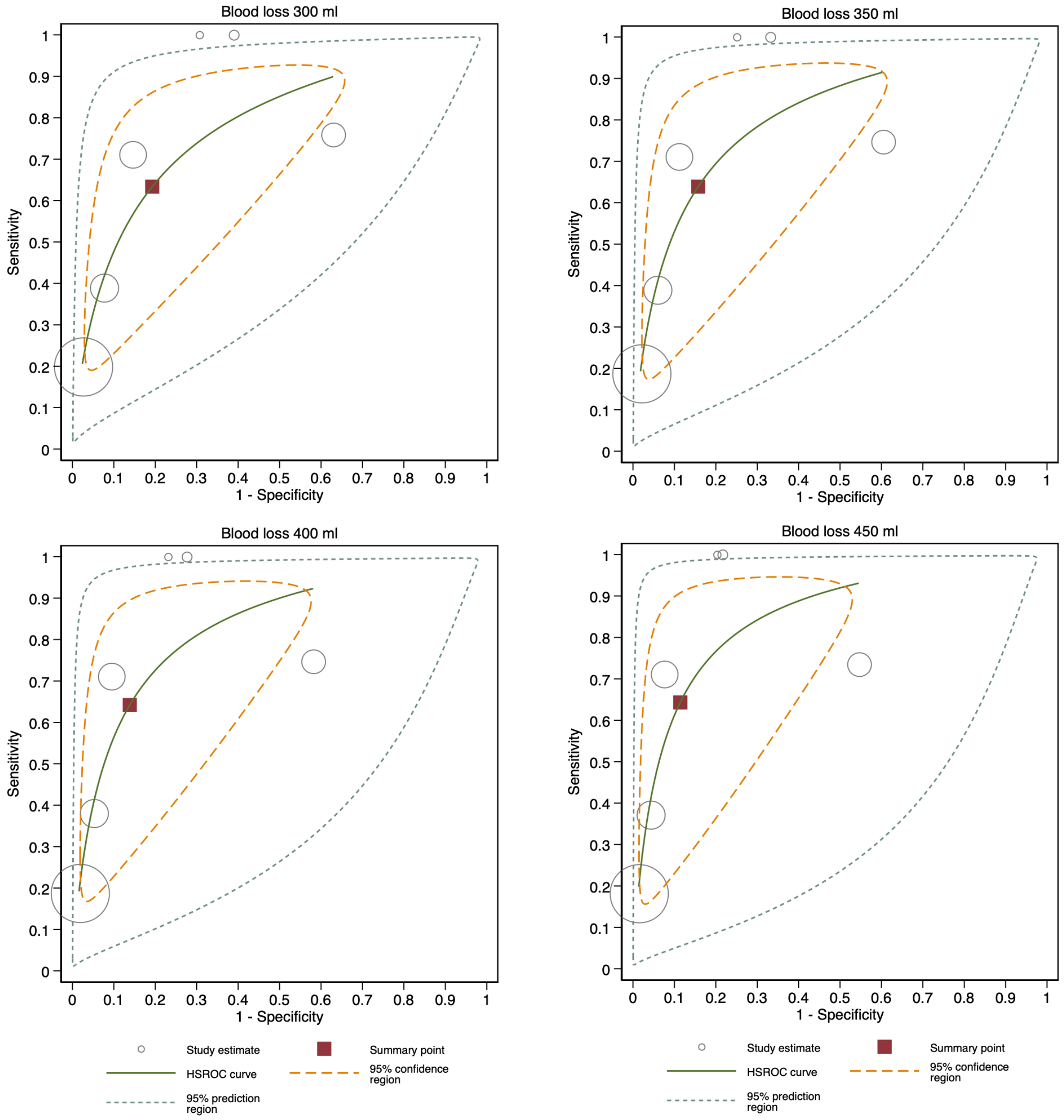

* Rules 1 to 3 are based on analysis of 30 691 women.

Figure S9. Summary ROC curve for blood loss thresholds in combination with haemodynamic signs in predicting maternal death or severe morbidity (Decision rule 3: Point system (≥2 points) (blood loss between lower threshold and 499 ml, pulse >100 bpm, systolic <100 mmHg, diastolic blood pressure <60 mmHg, shock index >1 = 1 point; blood loss ≥500 ml = 2 points)).

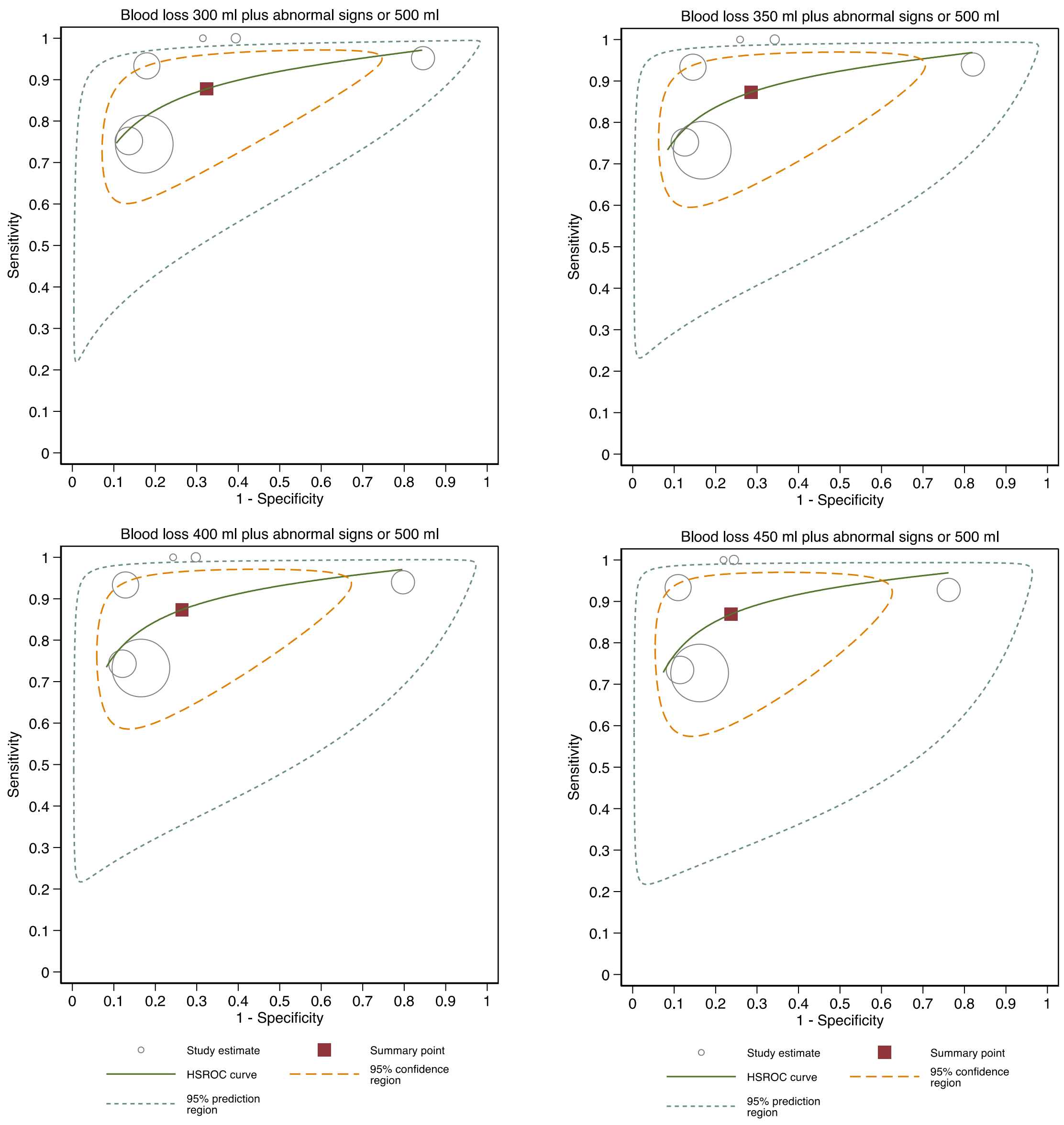

[*] Rules 1 to 3 are based on analysis of 30 691 women.

Figure S10. Subgroup analyses (by mode of birth, income, risk).

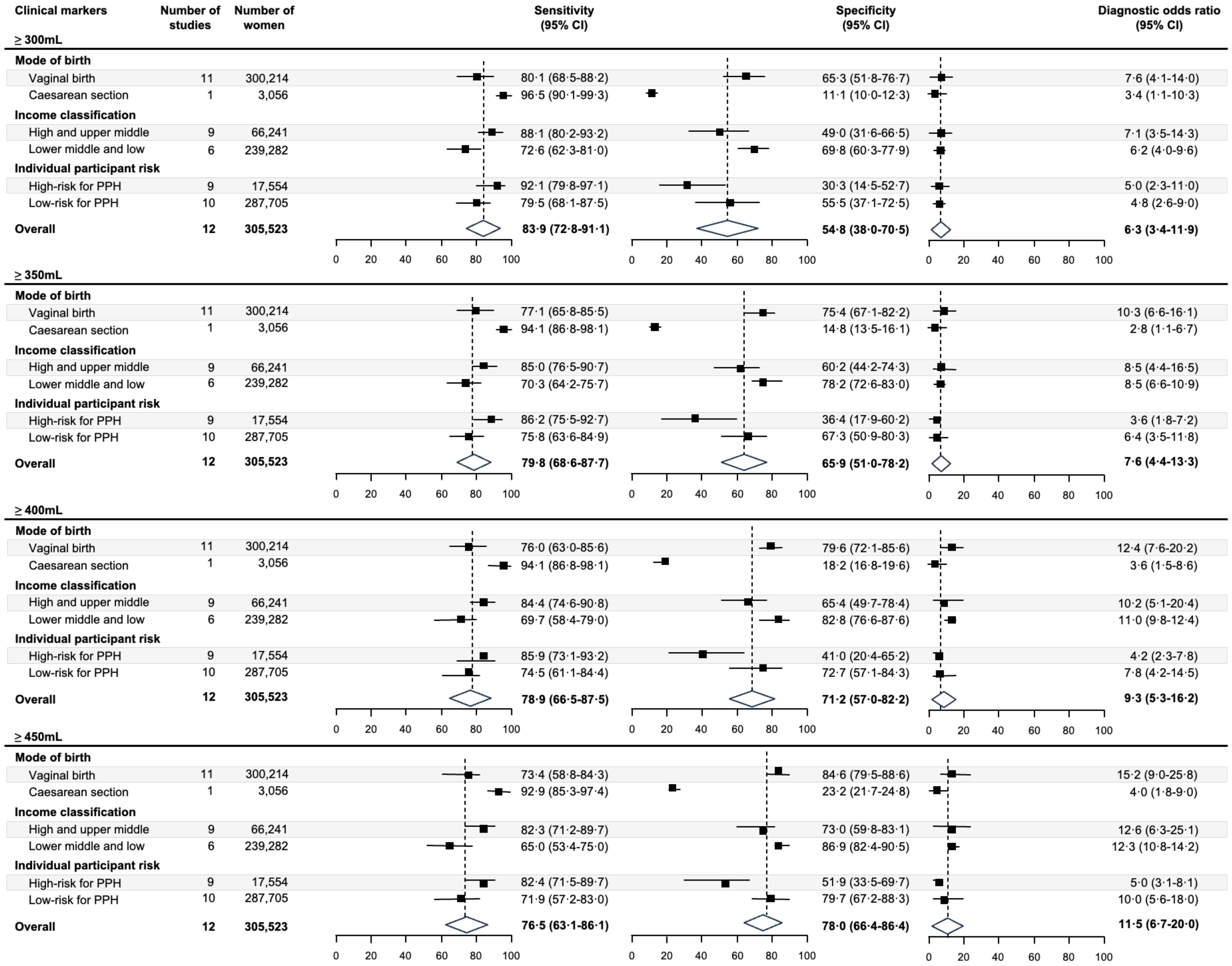

≥ 500mL

Clinical markers	Number of studies	Number of women	Sensitivity (95% CI)	Specificity (95% CI)	Diagnostic odds ratio (95% CI)
**Mode of birth**					
Vaginal birth	11	300,214	72·8 (56·7-84·6)	87·0 (82·1-90·7)	17·9 (10·1-31·9)
Caesarean section	1	3,056	92·9 (85·3-97·4)	27·4 (25·8-29·1)	5·0 (2·2-11·2)
**Income classification**					
High and upper middle	9	66,241	82·1 (70·4-89·9)	76·8 (64·2-85·9)	15·2 (7·3-31·7)
Lower middle and low	6	239,282	61·5 (50·0-71·8)	89·3 (85·3-92·3)	13·3 (11·9-15·0)
**Individual participant risk**					
High-risk for PPH	9	17,554	81·8 (68·9-90·1)	55·6 (36·6-73·1)	5·6 (3·6-8·8)
Low-risk for PPH	10	287,705	70·4 (53·3-83·2)	83·2 (71·8-90·6)	11·7 (5·7-24·0)
**Overall**	**12**	**305,523**	**75·7 (60·3-86·4)**	**81·4 (70·7-88·8)**	**13·6 (7·2-25·8)**

≥ 550mL

Clinical markers	Number of studies	Number of women	Sensitivity (95% CI)	Specificity (95% CI)	Diagnostic odds ratio (95% CI)
**Mode of birth**					
Vaginal birth	11	300,214	71·2 (53·7-84·0)	89·7 (86·0-92·5)	21·5 (11·9-38·9)
Caesarean section	1	3,056	88·2 (79·4-94·2)	31·8 (30·2-33·6)	3·5 (1·8-6·7)
**Income classification**					
High and upper middle	9	66,241	79·9 (68·0-88·1)	81·3 (70·8-88·6)	17·2 (8·0-37·0)
Lower middle and low	6	239,282	54·2 (41·4-66·5)	91·6 (88·4-94·0)	13·0 (8·3-20·2)
**Individual participant risk**					
High-risk for PPH	9	17,554	77·9 (65·3-86·8)	62·0 (43·5-77·5)	5·7 (3·7-8·9)
Low-risk for PPH	10	287,705	66·4 (48·8-80·4)	87·0 (77·9-92·7)	13·2 (6·2-28·0)
**Overall**	**12**	**305,523**	**72·4 (55·3-84·7)**	**85·3 (76·6-91·2)**	**15·2 (7·4-31·3)**

≥ 600mL

Clinical markers	Number of studies	Number of women	Sensitivity (95% CI)	Specificity (95% CI)	Diagnostic odds ratio (95% CI)
**Mode of birth**					
Vaginal birth	11	300,214	70·1 (51·9-83·6)	91·2 (87·8-93·7)	24·3 (13·5-44·0)
Caesarean section	1	3,056	84·7 (75·3-91·6)	36·4 (34·6-38·1)	3·6 (1·8-5·7)
**Income classification**					
High and upper middle	9	66,241	78·8 (66·7-87·3)	83·5 (73·9-90·1)	18·8 (8·5-41·4)
Lower middle and low	6	239,282	52·5 (39·3-65·3)	93·1 (90·3-95·1)	14·9 (9·8-22·6)
**Individual participant risk**					
High-risk for PPH	9	17,554	77·6 (63·8-87·2)	64·7 (46·2-79·7)	6·3 (4·1-9·8)
Low-risk for PPH	10	287,705	63·7 (46·2-78·2)	88·7 (80·4-93·8)	13·8 (6·3-30·4)
**Overall**	**12**	**305,523**	**70·9 (53·4-83·8)**	**87·3 (79·4-92·5)**	**16·8 (8·0-35·0)**

≥ 700mL

Clinical markers	Number of studies	Number of women	Sensitivity (95% CI)	Specificity (95% CI)	Diagnostic odds ratio (95% CI)
**Mode of birth**					
Vaginal birth	11	300,214	64·6 (46·3-79·5)	93·8 (90·9-95·8)	27·5 (16·5-45·9)
Caesarean section	1	3,056	81·2 (71·2-88·8)	45·4 (43·6-47·3)	3·6 (2·1-6·2)
**Income classification**					
High and upper middle	9	66,241	74·0 (61·7-83·3)	88·0 (80·5-92·9)	20·8 (10·1-42·9)
Lower middle and low	6	239,282	46·5 (33·8-59·7)	95·5 (93·5-96·9)	18·6 (12·4-27·8)
**Individual participant risk**					
High-risk for PPH	9	17,554	73·8 (56·6-85·9)	73·0 (56·3-85·1)	7·6 (4·5-12·9)
Low-risk for PPH	10	287,705	58·5 (40·4-74·5)	92·3 (86·2-95·9)	16·9 (7·9-36·2)
**Overall**	**12**	**305,523**	**64·8 (47·3-79·0)**	**91·1 (85·1-94·9)**	**18·9 (9·6-37·2)**

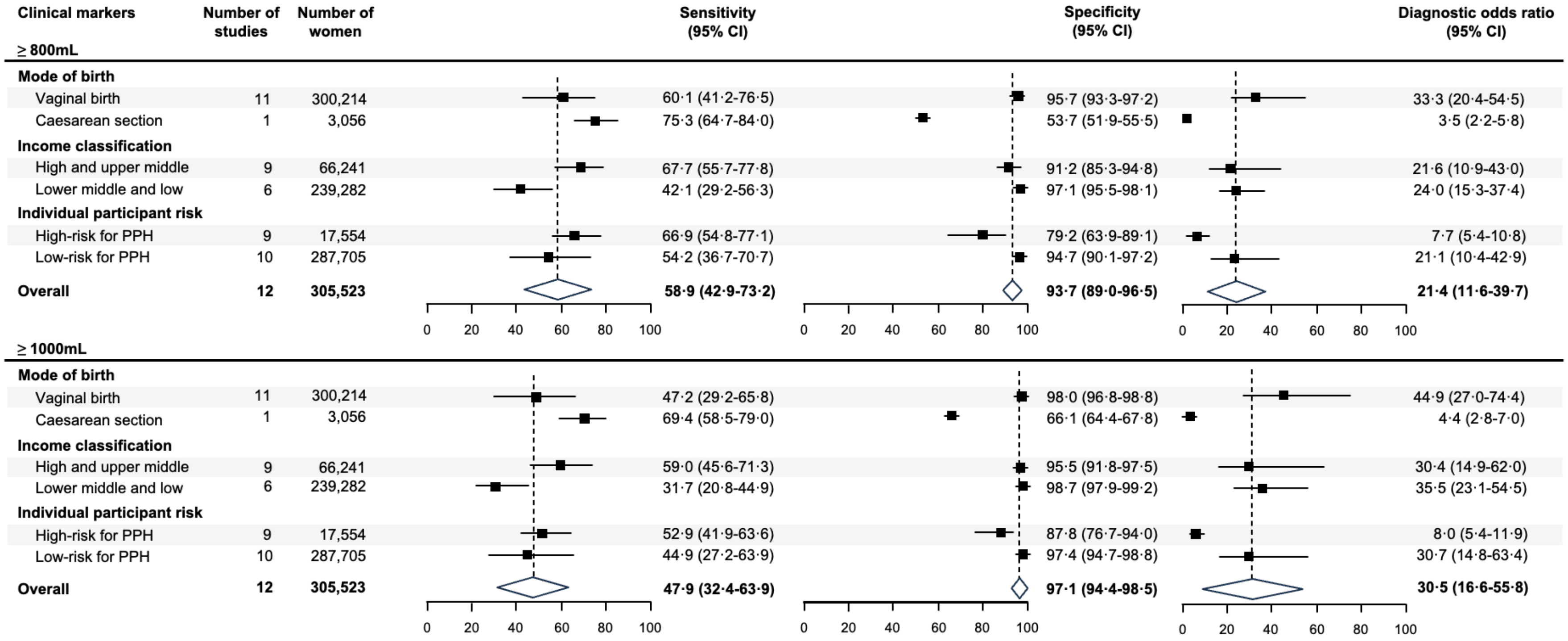

Clinical markers	Number of studies	Number of women	Sensitivity (95% CI)	Specificity (95% CI)	Diagnostic odds ratio (95% CI)
**≥ 800mL**					
**Mode of birth**					
Vaginal birth	11	300,214	60·1 (41·2-76·5)	95·7 (93·3-97·2)	33·3 (20·4-54·5)
Caesarean section	1	3,056	75·3 (64·7-84·0)	53·7 (51·9-55·5)	3·5 (2·2-5·8)
**Income classification**					
High and upper middle	9	66,241	67·7 (55·7-77·8)	91·2 (85·3-94·8)	21·6 (10·9-43·0)
Lower middle and low	6	239,282	42·1 (29·2-56·3)	97·1 (95·5-98·1)	24·0 (15·3-37·4)
**Individual participant risk**					
High-risk for PPH	9	17,554	66·9 (54·8-77·1)	79·2 (63·9-89·1)	7·7 (5·4-10·8)
Low-risk for PPH	10	287,705	54·2 (36·7-70·7)	94·7 (90·1-97·2)	21·1 (10·4-42·9)
**Overall**	**12**	**305,523**	**58·9 (42·9-73·2)**	**93·7 (89·0-96·5)**	**21·4 (11·6-39·7)**
**≥ 1000mL**					
**Mode of birth**					
Vaginal birth	11	300,214	47·2 (29·2-65·8)	98·0 (96·8-98·8)	44·9 (27·0-74·4)
Caesarean section	1	3,056	69·4 (58·5-79·0)	66·1 (64·4-67·8)	4·4 (2·8-7·0)
**Income classification**					
High and upper middle	9	66,241	59·0 (45·6-71·3)	95·5 (91·8-97·5)	30·4 (14·9-62·0)
Lower middle and low	6	239,282	31·7 (20·8-44·9)	98·7 (97·9-99·2)	35·5 (23·1-54·5)
**Individual participant risk**					
High-risk for PPH	9	17,554	52·9 (41·9-63·6)	87·8 (76·7-94·0)	8·0 (5·4-11·9)
Low-risk for PPH	10	287,705	44·9 (27·2-63·9)	97·4 (94·7-98·8)	30·7 (14·8-63·4)
**Overall**	**12**	**305,523**	**47·9 (32·4-63·9)**	**97·1 (94·4-98·5)**	**30·5 (16·6-55·8)**

Figures S11. Sensitivity Analyses (by treatment threshold)

Figure S11. Summary ROC curve for blood loss thresholds in predicting maternal death or severe morbidity (sensitivity analysis: women treated at the conventional ≥500 mL blood loss threshold).

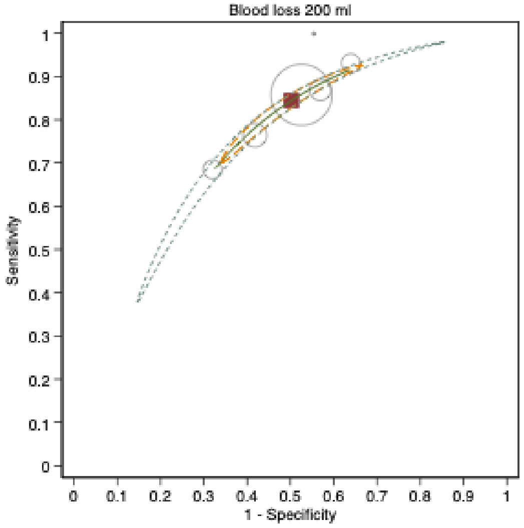

[*] Figures are based on analysis of 256 845 women.

Tables S3–S4: Prognostic Accuracy Estimates (for sensitivity analyses)

Table S3. Summary prognostic accuracy estimates for blood loss thresholds in predicting maternal death or severe morbidity (sensitivity analysis: women treated at the conventional ≥ 500 mL blood loss threshold only).

Threshold	Sensitivity	(95% CI)	Specificity	(95% CI)	DOR	(95% CI)	LR+	(95% CI)	LR-	(95% CI)
200 mL	90·0	(80·6 –95·1)	30·6	(16·0 –50·7)	4·0	(1·6 –10·1)	1·3	(1·0 –1·7)	0·3	(0·2 –0·7)
250 mL	86·2	(76·6 –92·3)	46·2	(30·5 –62·7)	5·4	(3·0 –9·7)	1·6	(1·2 –2·1)	0·3	(0·2 –0·5)
300 mL	84·1	(73·1 –91·1)	54·4	(37·8 –70·2)	6·3	(3·3 –11·8)	1·8	(1·3 –2·5)	0·3	(0·2 –0·5)
350 mL	80·0	(68·9 –87·8)	65·6	(50·8 –77·9)	7·6	(4·4 –13·2)	2·3	(1·6 –3·3)	0·3	(0·2 –0·5)
400 mL	79·1	(66·9 –87·7)	70·9	(56·7 –82·0)	9·2	(5·3 –16·2)	2·7	(1·9 –4·0)	0·3	(0·2 –0·4)
450 mL	76·7	(63·5 –86·2)	77·8	(66·2 –86·2)	11·5	(6·7 –20·0)	3·5	(2·4 –5·1)	0·3	(0·2 –0·5)
500 mL	75·9	(60·7 –86·5)	81·2	(70·5 –88·7)	13·6	(7·2 –25·8)	4·0	(2·7 –6·1)	0·3	(0·2 –0·5)
550 mL	72·6	(55·7 –84·9)	85·2	(76·4 –91·0)	15·2	(7·4 –31·3)	4·9	(3·2 –7·6)	0·3	(0·2 –0·5)
600 mL	71·2	(53·8 –83·9)	87·2	(79·2 –92·4)	16·8	(8·0 –35·0)	5·5	(3·5 –8·8)	0·3	(0·2 –0·5)
700 mL	65·1	(47·7 –79·2)	91·0	(84·9 –94·8)	18·9	(9·6 –37·2)	7·3	(4·5 –11·6)	0·4	(0·2 –0·6)
800 mL	59·2	(43·3 –73·4)	93·7	(88·9 –96·5)	21·4	(11·6 –39·7)	9·3	(5·7 –15·3)	0·4	(0·3 –0·6)
1 000 mL	48·3	(32·8 –64·1)	97·0	(94·3 –98·5)	30·4	(16·6 –55·6)	16·2	(9·5 –27·6)	0·5	(0·4 –0·7)

* Table is based on analysis of 256 845 women.

Table S4. Summary prognostic accuracy estimates for blood loss thresholds in predicting maternal death or severe morbidity (sensitivity analysis: women with weighed blood loss only).

**Threshold**	**Sensitivity**	**(95% CI)**	**Specificity**	**(95% CI)**	**DOR**	**(95% CI)**	**LR+**	**(95% CI)**	**LR-**	**(95% CI)**
200 mL	84·4	(77·7 –89·3)	49·8	(41·0 –58·5)	5·4	(4·8 –5·9)	1·7	(1·5 –1·9)	0·3	(0·3 –0·4)
250 mL	79·5	(71·6 –85·7)	62·6	(54·2 –70·3)	6·5	(5·5 –7·7)	2·1	(1·9 –2·4)	0·3	(0·3 –0·4)
300 mL	75·7	(67·4 –82·4)	70·3	(62·8 –76·9)	7·4	(6·5 –8·4)	2·6	(2·2 –3·0)	0·3	(0·3 –0·4)
350 mL	71·6	(62·9 –79·0)	77·8	(71·3 –83·1)	8·9	(7·8 –10·1)	3·2	(2·7 –3·8)	0·4	(0·3 –0·5)
400 mL	69·2	(59·3 –77·6)	81·9	(76·0 –86·5)	10·1	(8·8 –11·7)	3·8	(3·2 –4·5)	0·4	(0·3 –0·5)
450 mL	65·9	(54·8 –75·4)	85·8	(80·9 –89·6)	11·6	(10·0 –13·5)	4·6	(4·0 –5·4)	0·4	(0·3 –0·5)
500 mL		(52·1 –74·1)	88·1	(83·6 –91·4)	13·0	(11·5 –14·7)	5·3	(4·6 –6·2)	0·4	(0·3 –0·5)
550 mL	60·3	(48·1 –71·3)	90·5	(86·7 –93·2)	14·4	(12·7 –16·3)	6·3	(5·5 –7·3)	0·4	(0·3 –0·6)
600 mL	56·9	(45·2 –67·9)	92·1	(88·9 –94·4)	15·4	(13·9 –17·2)	7·2	(6·2 –8·3)	0·5	(0·4 –0·6)
700 mL	52·5	(38·8 –65·8)	94·6	(92·0 –96·4)	19·4	(15·8 –24·0)	9·8	(8·2 –11·6)	0·5	(0·4 –0·7)
800 mL	48·4	(33·6 –63·5)	96·4	(94·2 –97·7)	24·8	(18·9 –32·4)	13·3	(10·7 –16·4)	0·5	(0·4 –0·7)
1 000 mL	34·6	(22·9 –48·6)	98·5	(97·5 –99·1)	33·9	(24·5 –46·9)	22·5	(17·1 –29·6)	0·7	(0·5 –0·8)

* Table is based on analysis of 295 751 women

6. Supplementary Materials

PRISMA-IPD Checklist

PRISMA-IPD Section/topic	Item No	Checklist item	Reported on page
**Title**			
Title	1	Identify the report as a systematic review and meta-analysis of individual participant data.	Title #1
**Abstract**			
Structured summary	2	Provide a structured summary including as applicable:	Abstract #2 (some of this in main text due to word limit)
		**Background**: state research question and main objectives, with information on participants, interventions, comparators and outcomes.	
		**Methods**: report eligibility criteria; data sources including dates of last bibliographic search or elicitation, noting that IPD were sought; methods of assessing risk of bias.	
		**Results**: provide number and type of studies and participants identified and number (%) obtained; summary effect estimates for main outcomes (benefits and harms) with confidence intervals and measures of statistical heterogeneity. Describe the direction and size of summary effects in terms meaningful to those who would put findings into practice.	
		**Discussion:** state main strengths and limitations of the evidence, general interpretation of the results and any important implications.	
		**Other:** report primary funding source, registration number and registry name for the systematic review and IPD meta-analysis.	
**Introduction**			
Rationale	3	Describe the rationale for the review in the context of what is already known.	#4
Objectives	4	Provide an explicit statement of the questions being addressed with reference, as applicable, to participants, interventions, comparisons, outcomes and study design (PICOS). Include any hypotheses that relate to particular types of participant-level subgroups.	#4
**Methods**			
Protocol and registration	5	Indicate if a protocol exists and where it can be accessed. If available, provide registration information including registration number and registry name. Provide publication details, if applicable.	#4
Eligibility criteria	6	Specify inclusion and exclusion criteria including those relating to participants, interventions, comparisons, outcomes, study design and characteristics (e.g. years when conducted, required minimum follow-up). Note whether these were applied at the study or individual level i.e. whether eligible participants were included (and ineligible participants excluded) from a study that included a wider population than specified by the review inclusion criteria. The rationale for criteria should be stated.	#5

Identifying studies - information sources	7	Describe all methods of identifying published and unpublished studies including, as applicable: which bibliographic databases were searched with dates of coverage; details of any hand searching including of conference proceedings; use of study registers and agency or company databases; contact with the original research team and experts in the field; open adverts and surveys. Give the date of last search or elicitation.	#4-5, Supplementary methods in the appendix
Identifying studies - search	8	Present the full electronic search strategy for at least one database, including any limits used, such that it could be repeated.	#4, Supplementary methods in the appendix
Study selection processes	9	State the process for determining which studies were eligible for inclusion.	#4-5, Supplementary methods in the appendix
Data collection processes	10	Describe how IPD were requested, collected and managed, including any processes for querying and confirming data with investigators. If IPD were not sought from any eligible study, the reason for this should be stated (for each such study).	#4-5, Supplementary methods in the appendix
		If applicable, describe how any studies for which IPD were not available were dealt with. This should include whether, how and what aggregate data were sought or extracted from study reports and publications (such as extracting data independently in duplicate) and any processes for obtaining and confirming these data with investigators.	
Data items	11	Describe how the information and variables to be collected were chosen. List and define all study level and participant level data that were sought, including baseline and follow-up information. If applicable, describe methods of standardising or translating variables within the IPD datasets to ensure common scales or measurements across studies.	#5, Supplementary methods in the appendix
IPD integrity	A1	Describe what aspects of IPD were subject to data checking (such as sequence generation, data consistency and completeness, baseline imbalance) and how this was done.	Supplementary methods in the appendix
Risk of bias assessment in individual studies.	12	Describe methods used to assess risk of bias in the individual studies and whether this was applied separately for each outcome. If applicable, describe how findings of IPD checking were used to inform the assessment. Report if and how risk of bias assessment was used in any data synthesis.	#5, Supplementary methods in the appendix
Specification of outcomes and effect measures	13	State all treatment comparisons of interests. State all outcomes addressed and define them in detail. State whether they were pre-specified for the review and, if applicable, whether they were primary/main or secondary/additional outcomes. Give the principal measures of effect (such as risk ratio, hazard ratio, difference in means) used for each outcome.	#5, Supplementary methods in the appendix
Synthesis methods	14	Describe the meta-analysis methods used to synthesise IPD. Specify any statistical methods and models used. Issues should include (but are not restricted to):	#5-7, Supplementary methods in the appendix

		• Use of a one-stage or two-stage approach.<br>• How effect estimates were generated separately within each study and combined across studies (where applicable).<br>• Specification of one-stage models (where applicable) including how clustering of patients within studies was accounted for.<br>• Use of fixed or random effects models and any other model assumptions, such as proportional hazards.<br>• How (summary) survival curves were generated (where applicable).<br>• Methods for quantifying statistical heterogeneity (such as I^2 and t^2).<br>• How studies providing IPD and not providing IPD were analysed together (where applicable).<br>• How missing data within the IPD were dealt with (where applicable).	
Exploration of variation in effects	A2	If applicable, describe any methods used to explore variation in effects by study or participant level characteristics (such as estimation of interactions between effect and covariates). State all participant-level characteristics that were analysed as potential effect modifiers, and whether these were pre-specified.	#6, Supplementary methods in the appendix
Risk of bias across studies	15	Specify any assessment of risk of bias relating to the accumulated body of evidence, including any pertaining to not obtaining IPD for particular studies, outcomes or other variables.	#6, Supplementary methods in the appendix
Additional analyses	16	Describe methods of any additional analyses, including sensitivity analyses. State which of these were pre-specified.	#6-7, Supplementary methods in the appendix
**Results**			
Study selection and IPD obtained	17	Give numbers of studies screened, assessed for eligibility, and included in the systematic review with reasons for exclusions at each stage. Indicate the number of studies and participants for which IPD were sought and for which IPD were obtained. For those studies where IPD were not available, give the numbers of studies and participants for which aggregate data were available. Report reasons for non-availability of IPD. Include a flow diagram.	#7, Figure 1
Study characteristics	18	For each study, present information on key study and participant characteristics (such as description of interventions, numbers of participants, demographic data, unavailability of outcomes, funding source, and if applicable duration of follow-up). Provide (main) citations for each study. Where applicable, also report similar study characteristics for any studies not providing IPD.	#7, Table S1
IPD integrity	A3	Report any important issues identified in checking IPD or state that there were none.	#7, Supplementary methods in the appendix
Risk of bias within studies	19	Present data on risk of bias assessments. If applicable, describe whether data checking led to the up-weighting or down-weighting of these assessments. Consider how any potential bias impacts on the robustness of meta-analysis conclusions.	#7, Figure S1

Results of individual studies	20	For each comparison and for each main outcome (benefit or harm), for each individual study report the number of eligible participants for which data were obtained and show simple summary data for each intervention group (including, where applicable, the number of events), effect estimates and confidence intervals. These may be tabulated or included on a forest plot.	#7, Table S1
Results of syntheses	21	Present summary effects for each meta-analysis undertaken, including confidence intervals and measures of statistical heterogeneity. State whether the analysis was pre-specified, and report the numbers of studies and participants and, where applicable, the number of events on which it is based.	#7-8, Supplementary results in the Appendix
		When exploring variation in effects due to patient or study characteristics, present summary interaction estimates for each characteristic examined, including confidence intervals and measures of statistical heterogeneity. State whether the analysis was pre-specified. State whether any interaction is consistent across trials.	
		Provide a description of the direction and size of effect in terms meaningful to those who would put findings into practice.	
Risk of bias across studies	22	Present results of any assessment of risk of bias relating to the accumulated body of evidence, including any pertaining to the availability and representativeness of available studies, outcomes or other variables.	#7, Figure 1
Additional analyses	23	Give results of any additional analyses (e.g. sensitivity analyses). If applicable, this should also include any analyses that incorporate aggregate data for studies that do not have IPD. If applicable, summarise the main meta-analysis results following the inclusion or exclusion of studies for which IPD were not available.	#7-8, Supplementary results in the Appendix
**Discussion**			
Summary of evidence	24	Summarise the main findings, including the strength of evidence for each main outcome.	#8-9
Strengths and limitations	25	Discuss any important strengths and limitations of the evidence including the benefits of access to IPD and any limitations arising from IPD that were not available.	#9-10
Conclusions	26	Provide a general interpretation of the findings in the context of other evidence.	#10-11
Implications	A4	Consider relevance to key groups (such as policy makers, service providers and service users). Consider implications for future research.	#10
**Funding**			
Funding	27	Describe sources of funding and other support (such as supply of IPD), and the role in the systematic review of those providing such support.	#7 and 12-13

A1 – A3 denote new items that are additional to standard PRISMA items. A4 has been created as a result of re-arranging content of the standard PRISMA statement to suit the way that systematic review IPD meta-analyses are reported.

© Reproduced with permission of the PRISMA IPD Group, which encourages sharing and reuse for non-commercial purposes.

Consortium Members and Collaborators

Members of the *WHO Consortium on Postpartum Haemorrhage Definition* (listed in alphabetical order within each group) include:

**Lead investigators and country principal investigators of the original studies**		
Anderson	BOROVAC-PINHEIRO	University of Campinas, São Paulo, Brazil
Guillermo	CARROLI	Centro Rosarino de Estudios Perinatales, Rosario, Argentina
Arri	COOMARASAMY	Tommy's National Centre for Miscarriage Research, University of Birmingham, UK
Jill	DUROCHER	Gynuity Health Projects, New York, USA
Fadhlun M.	ALWY AL-BEITY	Muhimbili University of Health and Allied Sciences, Dar es Salaam, Tanzania
Sue	FAWCUS	University of Cape Town, South Africa
Mario	FESTIN	University of the Philippines, Manila, The Philippines
Hadiza S.	GALADANCI	Bayero University, Kano, Nigeria
Shivaprasad	GOUDAR	Women's and Children's Health Research Unit, Jawaharlal Nehru Medical College, KLE Academy of Higher Education and Research, Belagavi, India
A. Metin	GÜLMEZOGLU	Concept Foundation, Geneva, Switzerland
Christian	HASLINGER	Department of Obstetrics, University Hospital Zurich, University of Zurich, Switzerland
G. Justus	HOFMEYR	Effective Care Research Unit, University of the Witwatersrand, Walter Sisulu University, East London, South Africa
Pisake	LUMBIGANON	Faculty of Medicine, Khon Kaen University, Thailand
Kidza	MUGERWA	Makerere University, Kampala, Uganda
Rodolfo C.	PACAGNELLA	University of Campinas, São Paulo, Brazil
Zahida	QURESHI	University of Nairobi, Nairobi, Kenya
Loïc	SENTILHES	Department of Obstetrics and Gynecology, Bordeaux University Hospital, France
Lumaan	SHEIKH	Aga Khan University, Karachi, Pakistan
**Members of the PPH Definition Evidence Synthesis Team**		
John	ALLOTEY	Institute of Life Course and Medical Sciences, University of Liverpool, Liverpool, UK
Arri	COOMARASAMY	Tommy's National Centre for Miscarriage Research, University of Birmingham, UK
Adam	DEVALL	Tommy's National Centre for Miscarriage Research, University of Birmingham, UK
Jonathan J.	DEEKS	Department of Applied Health Sciences, University of Birmingham, Birmingham, UK
Malcolm	PRICE	Department of Public Health, Canadian University Dubai, Dubai, United Arab Emirates
Soha	SOBHY	Tommy's National Centre for Miscarriage Research, University of Birmingham, UK
Aurelio	TOBIAS	Spanish Council for Scientific Research, Barcelona, Spain

Idnan	YUNAS	Department of Metabolism and Systems Science, College of Medicine and Health, University of Birmingham, Birmingham, UK
**WHO Project Secretariat**		
Fernando	ALTHABE	UNDP–UNFPA–UNICEF–WHO–World Bank Special Program of Research, Development, and Research Training in Human Reproduction, Department of Sexual and Reproductive Health and Research, WHO, Geneva, Switzerland
Jenny	CRESSWELL	UNDP–UNFPA–UNICEF–WHO–World Bank Special Program of Research, Development, and Research Training in Human Reproduction, Department of Sexual and Reproductive Health and Research, WHO, Geneva, Switzerland
Ioannis	GALLOS	UNDP–UNFPA–UNICEF–WHO–World Bank Special Program of Research, Development, and Research Training in Human Reproduction, Department of Sexual and Reproductive Health and Research, WHO, Geneva, Switzerland
Olufemi T.	OLADAPO	UNDP–UNFPA–UNICEF–WHO–World Bank Special Program of Research, Development, and Research Training in Human Reproduction, Department of Sexual and Reproductive Health and Research, WHO, Geneva, Switzerland
Caitlin R.	WILLIAMS	UNDP–UNFPA–UNICEF–WHO–World Bank Special Program of Research, Development, and Research Training in Human Reproduction, Department of Sexual and Reproductive Health and Research, WHO, Geneva, Switzerland

7. References

1. Pritchard JA., Baldwin RM, Dickey JC, Wiggins KM, Reed GP, Bruce DM. Blood volume changes in pregnancy and the puerperium. Am J Obstet Gynecol. 1962; 84(10):1271–82.
2. de Vries PLM, Deneux‐Tharaux C, Baud D, Chen KK, Donati S, Goffinet F, et al. Postpartum haemorrhage in high‐resource settings: Variations in clinical management and future research directions based on a comparative study of national guidelines. BJOG. 2023; 130(13):1639-1652.
3. Meher S, Cuthbert A, Kirkham JJ, Williamson P, Abalos E, Aflaifel N, et al. Core outcome sets for prevention and treatment of postpartum haemorrhage: an international Delphi consensus study. BJOG. 2019; 126(1):83-9
4. Weeks J, Cuthbert A, Alfirevic Z. Trustworthiness assessment as an inclusion criterion for systematic reviews—What is the impact on results? Cochrane Ev Synth. 2023; e12037.
5. Lee J, Mulder F, Leeflang M, Wolff R, Whiting P, Bossuyt PM. QUAPAS: An Adaptation of the QUADAS-2 Tool to Assess Prognostic Accuracy Studies. Ann Intern Med. 2022; 175(7):1010-1018.
6. Stewart LA, Clarke M, Rovers M, Riley RD, Simmonds M, Stewart G, Tierney JF; PRISMA-IPD Development Group. Preferred Reporting Items for Systematic Review and Meta-Analyses of individual participant data: the PRISMA-IPD Statement. JAMA. 2015; 313(16):1657-1665.
7. Harbord RM, Whiting P. Metandi: Meta-analysis of Diagnostic Accuracy Using Hierarchical Logistic Regression. The Stata Journal. 2009; 9(2), 211-229.
8. Macaskill P, Takwoingi Y, Deeks JJ, Gatsonis C. Chapter 9: Understanding meta-analysis. In: Deeks JJ, Bossuyt PM, Leeflang MM, Takwoingi Y (editors). Cochrane Handbook for Systematic Reviews of Diagnostic Test Accuracy. Version 2.0 (updated July 2023). Cochrane, 2023. Available from https://training.cochrane.org/handbook-diagnostic-test-accuracy/current
